# Verwey-Type Charge Ordering and Site-Selective Mott Transition in Fe_4_O_5_ under Pressure

**DOI:** 10.1021/jacs.2c00895

**Published:** 2022-06-01

**Authors:** Samar Layek, Eran Greenberg, Stella Chariton, Maxim Bykov, Elena Bykova, Dmytro M. Trots, Alexander V. Kurnosov, Irina Chuvashova, Sergey V. Ovsyannikov, Ivan Leonov, Gregory Kh. Rozenberg

**Affiliations:** † School of Physics and Astronomy, Tel Aviv University, 69978 Tel Aviv, Israel; ‡ Department of Physics, School of Engineering, University of Petroleum and Energy Studies (UPES), Dehradun, Uttarakhand 248007, India; § Center for Advanced Radiation Sources, University of Chicago, 5640 South Ellis Avenue, 60637 Chicago, United States; ∥ Applied Physics Division, Soreq NRC, Yavne, 81800, Israel; ⊥ Institute of Inorganic Chemistry, University of Cologne, Greinstrasse 6, 50939 Cologne, Germany; # Earth and Planets Laboratory, Carnegie Institution for Science, Washington, District of Columbia 20015, United States; + Bayerisches Geoinstitut, Universität Bayreuth, Universitätsstrasse 30, D-95447 Bayreuth, Germany; ⊗ Harvard Physics, Jefferson Physical Lab, 17 Oxford Street, Cambridge, Massachusetts 02138, United States; × Department of Chemistry and Biochemistry, Florida International University, 11200 SW Eighth Street, CP 234, Miami, Florida 33199, United States; $ M. N. Miheev Institute of Metal Physics, Russian Academy of Sciences, 620108 Yekaterinburg, Russia; ¶ Ural Federal University, 620002 Yekaterinburg, Russia; ○ Skolkovo Institute of Science and Technology, 143026 Moscow, Russia

## Abstract

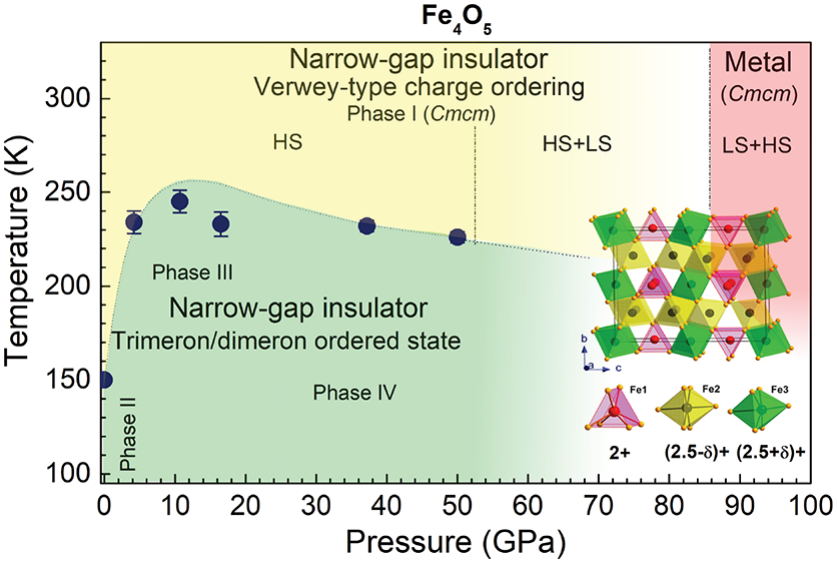

The metal–insulator transition driven by electronic correlations is one of the most fundamental concepts in condensed matter. In mixed-valence compounds, this transition is often accompanied by charge ordering (CO), resulting in the emergence of complex phases and unusual behaviors. The famous example is the archetypal mixed-valence mineral magnetite, Fe_3_O_4_, exhibiting a complex charge-ordering below the Verwey transition, whose nature has been a subject of long-time debates. In our study, using high-resolution X-ray diffraction supplemented by resistance measurements and DFT+DMFT calculations, the electronic, magnetic, and structural properties of recently synthesized mixed-valence Fe_4_O_5_ are investigated under pressure to ∼100 GPa. Our calculations, consistent with experiment, reveal that at ambient conditions Fe_4_O_5_ is a narrow-gap insulator characterized by the original Verwey-type CO. Under pressure Fe_4_O_5_ undergoes a series of electronic and magnetic-state transitions with an unusual compressional behavior above ∼50 GPa. A site-dependent collapse of local magnetic moments is followed by the site-selective insulator-to-metal transition at ∼84 GPa, occurring at the octahedral Fe sites. This phase transition is accompanied by a 2+ to 3+ valence change of the prismatic Fe ions and collapse of CO. We provide a microscopic explanation of the complex charge ordering in Fe_4_O_5_ which “unifies” it with the behavior of two archetypal examples of charge- or bond-ordered materials, magnetite and rare-earth nickelates (RNiO_3_). We find that at low temperatures the Verwey-type CO competes with the “trimeron”/“dimeron” charge ordered states, allowing for pressure/temperature tuning of charge ordering. Summing up the available data, we present the pressure–temperature phase diagram of Fe_4_O_5_.

## 1 Introduction

The electronic and magnetic transitions in strongly correlated transition metal oxides have been among the main topics of materials science and condensed matter physics over the last several decades.^1−4^ In such materials, the complex interplay between electronic correlations and the spin, charge, orbital, and lattice degrees of freedom leads to a wealth of ordering phenomena and complex phases, which makes these compounds highly attractive for technological applications.^1−4^ While being Mott or charge-transfer insulators at ambient pressure, these materials often exhibit a pressure-induced Mott insulator-to-metal phase transition (IMT), complicated by a spin-state crossover, collapse of magnetic moments and unit-cell volume, and suppression of charge and orbital ordering (in the charge and/or orbitally ordered compounds). In this respect basic iron oxides wüstite (FeO), hematite (Fe_2_O_3_), and mixed-valence magnetite (Fe_3_O_4_), the archetypal correlated insulating materials, have attracted much attention due to their complex electronic, magnetic, and structural behaviors.^2^

It was recently shown that under pressure, iron oxides demonstrate unexpected complexity of their compositions, such as FeO_2_, Fe_4_O_5_, Fe_5_O_6_, Fe_5_O_7_, Fe_7_O_9_, etc., with unusual crystal structures and complex electronic and magnetic properties,^5−15^ which can be systematized by homologous structural series *n*FeO·*m*Fe_2_O_3_ (with the exception of FeO_2_).^13^ Moreover, recent high-pressure (HP) studies of Fe_2_O_3_ and Fe_3_O_4_ suggest that these materials undergo a site-selective Mott transition, accompanied by a site-dependent collapse of magnetic moments.^16−18^ In both cases it goes along with site-dependent delocalization (metallization) of the 3d electrons. As a result, the high-spin to low-spin (HS-LS) state crossover and metallization at crystallographically different Fe sites occur at different pressures. Interestingly, in Fe_2_O_3_ the site-selective Mott transition is accompanied by a charge disproportionation of Fe ions, with Fe^(3±δ)+^ and δ ∼0.05–0.09.^17^ We note that of specific interest is the case of mixed-valence compounds, such as the archetypal mixed-valence material Fe_3_O_4_, which exhibits a complex charge ordering (CO) below the Verwey metal–insulator transition at *T*_V_ ∼ 122 K.^19−23^ While being intensively studied, the Verwey CO transition in Fe_3_O_4_ is still a subject of debates and the mechanism of the Verwey transition remains controversial.^19−31^ Only recently it was shown that Verwey’s hypothesis of CO of the Fe 2+ and 3+ ions below *T*_V_ in a regular pattern caused by a minimum of the electrostatic repulsion is correct to a first approximation. In fact, the localized Fe 3d electrons are proposed to be distributed over a linear arrangement of three octahedral Fe units, called “trimerons”.^22^ This behavior suggests the complex interplay between electronic correlations and the lattice in the low-temperature CO phase of Fe_3_O_4_.

**Figure 1:**
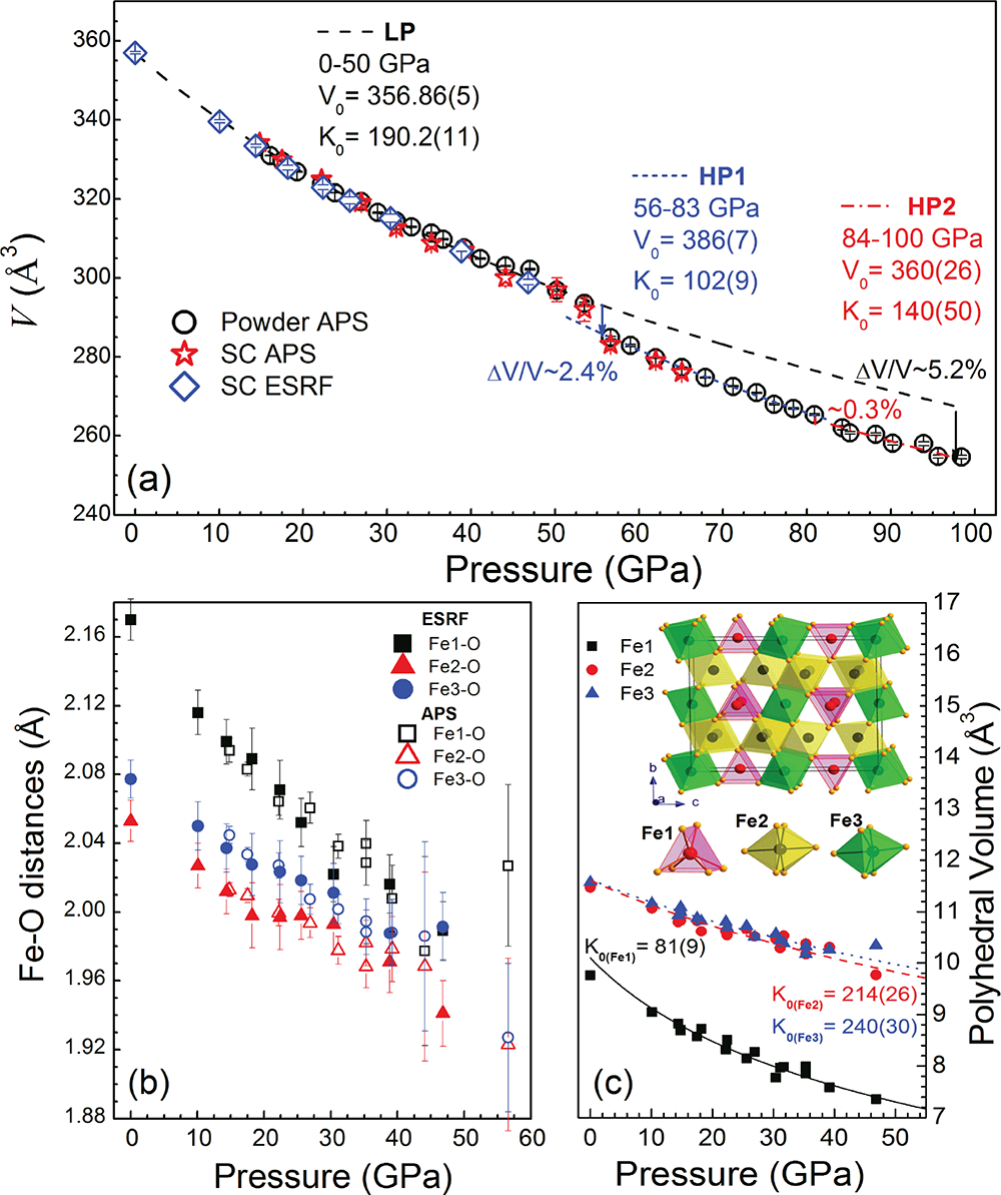
Compressional behavior of Fe_4_O_5_: (a) unit-cell volume, (b) average Fe–O distances, and (c) polyhedral volumes for different crystallographic sites of Fe_4_O_5_ as a function of pressure. The solid, dashed, dashed-dotted, and short-dashed lines in part a are fits with the second-order Birch–Murnaghan equation of state (BM2 EOS) (see the Supporting Information). Extrapolation of the low-pressure *V*(*P*) to higher pressures gives a volume drop Δ*V*/*V* ∼5.2% at ∼100 GPa. The solid, dashed, and dotted lines in part c are fits with the BM2 EOS for the Fe1, Fe2, and Fe3 sites, respectively. In the inset, we show the crystal structure of Fe_4_O_5_.

In our study, we focus on a recently discovered mixed-valence oxide Fe_4_O_5_, which can be synthesized at moderate pressure–temperature conditions and is found to be recoverable at ambient pressure.^5−8^ It was previously shown that Fe_4_O_5_ crystallizes in an orthorhombic CaFe_3_O_5_-type crystal structure (space group *Cmcm*), which is comprised of chains of trigonal prisms (Fe1 site), filled with Fe^2+^ cations and an octahedral network consisting of single and double chains of octahedra (Fe2 and Fe3 sites, respectively), whose sites are occupied by mixed Fe^2+^/Fe^3+^ cations^5−8^ (see Figure 1c and Supporting Information, Figure S1). In close similarity to magnetite, Fe_4_O_5_ exhibits a complex CO transition at low temperature below ∼150 K, involving different dimeric and trimeric CO states within the chains of Fe ions,^32^ with a high sensitivity of the CO pattern and ordering temperature to applied pressures.^7,8^ Interestingly, the parent system CaFe_3_O_5_ also exhibits a complex trimeron CO below room temperature (RT).^33^ Nonetheless, the electronic properties of Fe_4_O_5_ and, in particular, the interplay of the electronic structure, spin and CO states, and phase stability of Fe_4_O_5_ under pressure are still poorly understood, and the mechanism behind the complex CO behaviors of Fe_4_O_5_ under pressure is unknown. Moreover, we also note that the high-pressure crystal chemistry of iron-bearing compounds is highly relevant for geoscience and for understanding extra- and terrestrial minerals, where the disproportionation reaction may have played a key role in redox processes and the evolution of Earth (see ref (34) and references therein).

We explore the electronic structure, magnetic, and structural properties of Fe_4_O_5_ under HP to ∼100 GPa using high-resolution single-crystal and powder synchrotron X-ray diffraction (XRD) combined with resistance measurements and the DFT + dynamical mean-field theory (DFT+DMFT) electronic structure calculations.^35^ With DFT+DMFT, it becomes possible to capture all generic aspects of the interplay between the electronic correlations, magnetic states, and crystal structure of paramagnetic (PM) Fe_4_O_5_ under pressure.^16,17,35−37^ Our combined experimental and theoretical results give a microscopic understanding of the complex pressure-induced evolution of the electronic, magnetic, and structural properties of Fe_4_O_5_. We show that at ambient conditions, Fe_4_O_5_ exhibits a narrow-gap Mott insulating state associated with the “classical” Verwey-type CO of Fe^2+^/Fe^3+^ cations at the crystallographically distinct Fe sites. Under pressure, it exhibits a series of electronic transitions characterized by a site-dependent collapse of local moments above ∼50 GPa and site-selective Mott insulator–metal transition at ∼84 GPa, corroborating with a collapse of charge-ordering. We explain the complex CO in Fe_4_O_5_ which “unifies” it with the behavior of charge-ordered magnetite, Fe_3_O_4_. Our results suggest that the Verwey-type CO competes with the trimeron/dimeron charge ordered states at low temperatures, allowing for pressure and temperature tuning of CO in Fe_4_O_5_.

## 2 Materials and Methods

We perform high-resolution single-crystal and powder synchrotron XRD study of Fe_4_O_5_ samples synthesized at Bayerisches Geoinstitut, Bayreuth (Germany).^38^ Symmetric membrane and custom diamond anvil cells (DACs) were used to induce high pressure, with neon as a pressure-transmitting medium. Pressure was determined using the ruby *R*1 fluorescence line as a pressure marker, and the Ne and gold unit-cell volumes in the case of X-ray diffraction studies. Single-crystal (SC) and powder XRD experiments were performed at the ID15B beamline at the European Synchrotron Radiation Facility (ESRF), Grenoble, France, and at the 13-ID-D beamline of the Advanced Photon Source (APS), Argonne National Laboratory (Argonne, IL). Electrical resistance measurements were performed as a function of pressure and temperature using a standard four-probe method.

We supplement our experimental results with the DFT+DMFT electronic structure calculations of PM Fe_4_O_5_ under pressure.^35^ In DMFT a quantum many-body lattice problem is mapped onto a multiorbital quantum impurity model which is solved within a path integral formalism using, e.g., quantum Monte Carlo techniques.^35^ This allows us to treat on the same footing the spin, charge, orbital, and temperature dependent interactions of the d or f electrons which are quantified by an impurity self-energy, which is defined on the Matsubara frequency domain. We use the DFT+DMFT method implemented with full self-consistency over the charge density^36,37^ to compute the electronic structure, magnetic properties, and spin and valence configurations of iron of Fe_4_O_5_. In our DFT+DMFT calculations, we construct a basis set of atomic-centered symmetry-constrained Wannier functions for the partially occupied Fe 3d bands evaluated within DFT.^39^ The DFT+DMFT calculations are performed in the local basis set determined by diagonalization of the corresponding Fe 3d occupation matrices. We adopt the crystal structure parameters taken from single crystal XRD and compute the spectral properties, Fe 3d Wannier orbital occupancies, and local magnetic moments of PM Fe_4_O_5_ for the three distinct pressure regimes: at ∼39, 62, and 84 GPa. In our calculations, we employ the DFT+DMFT method implemented with plane-wave pseudopotentials^36^ with a continuous-time hybridization-expansion quantum Monte Carlo (segment) algorithm in DMFT.^40^ We use the Hubbard *U* = 6 eV and Hund’s exchange *J* = 0.89 eV, in accordance with previous estimates.^16,17,36^ In addition, we perform DFT+DMFT calculations with a smaller Hubbard parameter *U* = 5 eV in order to check the stability of the obtained results. In our DFT+DMFT calculations, the Coulomb interaction was treated within the density–density approximation neglecting spin-flip and pair hopping terms (which are generally assumed to be small) in the multiorbital Hubbard Hamiltonian. We also neglect the spin–orbit coupling. In DFT, we use the generalized gradient Perdew–Burke–Ernzerhof approximation for the correlation exchange functional.^41^ For further details on the experimental and theoretical methods, see the Supporting Information.

## 3 Results

### 3.1 XRD Measurements and Compressional Behavior

We begin with a combined high-resolution single-crystal and powder synchrotron XRD study of RT Fe_4_O_5_. Our results for the compressional behavior of Fe_4_O_5_ are summarized in Figure 1. In agreement with previous experiments, at ambient conditions Fe_4_O_5_ crystallizes in the orthorhombic *Cmcm* crystal structure^5−8^ (see Figure S1). We observe that the *Cmcm* phase persists up to ∼100 GPa (see Figures S2–S5 and Table S1). In particular, no new diffraction spots due to lowering of the *Cmcm* crystal symmetry were observed in the single-crystal XRD with pressure increase up to ∼94 GPa at RT (see Figures S2 and S3). Most notably, under pressure, we obtain two consecutive first-order phase transitions at ∼50 and 84 GPa, which are characterized by a collapse of the unit-cell volume of ∼2.4% and 0.3%, respectively, and by a drastic change of compressional behavior. We note that while the volume collapse at ∼50 GPa is obvious from both powder and SC XRD data, the structural changes at ∼84 GPa are subtle, with our thorough analysis of the powder XRD data detecting a volume anomaly of ∼1 Å^3^, which exceeds the error estimates according to GSAS-II of up to ∼0.5 Å^3^.^42^ In addition, our results for the compressional behavior and the electronic transition observed within this pressure range in our resistance measurements (see below) clearly indicate a robust phase transformation at ∼84 GPa.

The bulk modulus of the LP phase *K*_0_ ≃ 190 GPa, which is within the range of that for FeO (Fe^2+^) and Fe_2_O_3_ (Fe^3+^) at ambient pressure,^13,16,24,43−45^ unexpectedly drops to ∼102 GPa in the HP1 phase. At 84 GPa, there is an apparent slight increase of *K*_0_ to ∼140 GPa upon the transition to the HP2 phase (see Figure 1 and Table S2). Nonetheless, the latter value is remarkably over 20% lower than that of the LP phase, implying a highly unusual compressional behavior of Fe_4_O_5_. We note that at 50 GPa, the bulk modulus is *K*_50_ = 375(1) and 279(11) GPa for the LP and the HP1 phases, respectively, and at ∼84 GPa it is *K*_84_ = 388(11) and 430(60) GPa for the HP1 and HP2 phases, respectively (see also Table S2). Thus, for both types of analysis, the trend of the bulk modulus change is the same, implying anomalous softening of the lattice above ∼50 GPa.^46^ Moreover, we note a significant difference in the compressibility of the Fe sites at the 0–45 GPa range, which is likely related to the different types of oxygen coordination of the prismatic Fe1 and the octahedral Fe2 and Fe3 sites. It leads to the different Fe–O bonding and significantly different crystal-field splitting of the Fe 3d electronic states.

Since no symmetry change is observed in our XRD up to high pressures, we conclude that the phase transitions at 50 and 84 GPa are of electronic origin. Moreover, an unexpected softening of the lattice at ∼50 GPa, suggests that this phase transition is not related to a Mott IMT, which in contrast usually leads to a sizable increase of bulk modulus upon metallization.^2,36^ It is interesting to note that other iron oxides, FeO, Fe_2_O_3_, and Fe_3_O_4_ also reveal phase transitions which occur under a pressure of several dozen to 100 GPa.^13,16−18,36,43,44^ However, in contrast to Fe_4_O_5_, the phase transitions in those systems are usually accompanied by a symmetry change.

Under pressure, we find a continuous decrease of the Fe–O distances, with indications of the reduced compressibility of the FeO_6_ octahedra comprising the Fe2 sites at about the 20–35 GPa range (see Figure 1b). This feature coincides with an unusual compressional behavior of the lattice parameter *b* indicative of a high axial compression anisotropy of the LP phase. Namely, while parameters *a* and *c* show a similar behavior and almost do not exhibit any peculiarities over the entire pressure range, the parameter *b* shows a higher stiffness followed by a sudden drop around 50 GPa (see Figure S6). Only above ∼56 GPa, the *b* axis behaves similar to two other lattice parameters indicating the collapse of the axial anisotropy. This anomalous behavior can be tentatively attributed to an antiferromagnetic spin alignment at the Fe2 sites along the *b* axis (for details see the Supporting Information). The following remarkable shrinkage of the *b* axis and Fe2–O distances at around 50 GPa (see Figure 1b and Figure S6) suggests that the phase transition at ∼50 GPa is accompanied by a change of the electronic state of the Fe2 sites. Below we will show that it is indeed related to a HS–LS state crossover at the Fe2 sites.

Our bond valence sum (BVS) analysis^47^ suggests that the trigonal Fe1 sites are filled with Fe^2+^ cations, while the octahedral Fe2 and Fe3 sites are occupied by mixed-valence Fe^2+^/Fe^3+^ states, consistent with previous estimates.^7,8^ We note, however, that BVS has a limited sensitivity and understanding of the valence states of Fe_4_O_5_ requires a microscopic modeling of its electronic structure. In particular, a mixed valence state of the octahedral Fe2 and Fe3 sites proposed by BVS would imply metallic behavior of Fe_4_O_5_ at RT, similar to a metallic state of Fe_3_O_4_ for *T* > *T*_V_.^18−21,24^ As we will show below, this assumption contradicts our resistance measurements and the DFT+DMFT calculations.

### 3.2 Resistance Measurements

**Figure 2:**
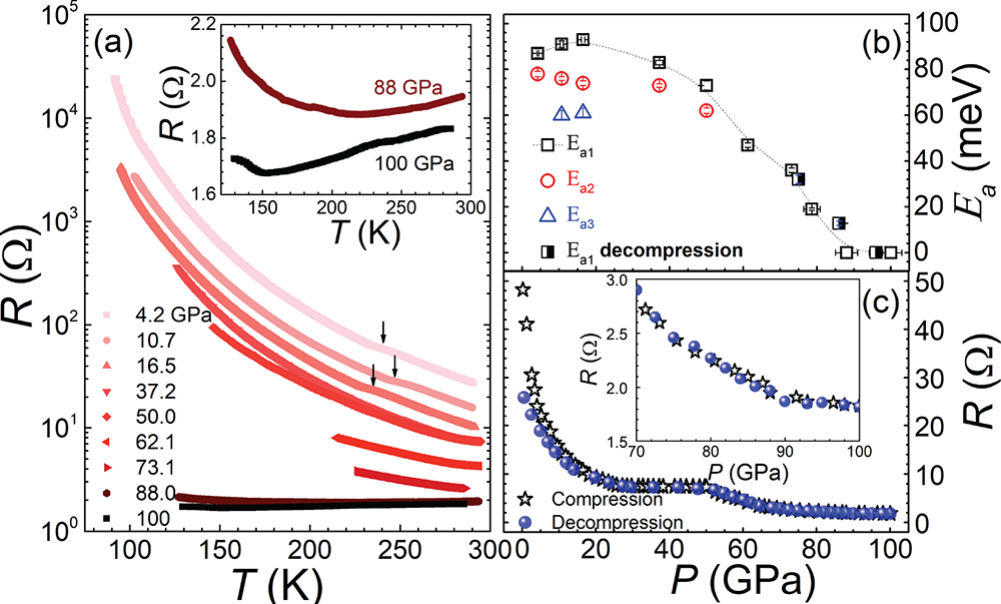
(a) Temperature-dependent *R*(*T*) resistance measured at various pressures. A part of the *R*(*T*) plot is expanded in the inset to emphasize the change of the *R*(*T*) slope sign with a temperature decrease at 88 and 100 GPa. (b) Pressure dependence of electrical transport activation energy for different temperature ranges: 290–240 K (*E*_a1_), 240–130 K (*E*_a2_), and 130–195 K (*E*_a3_), coinciding with different phases of Fe_4_O_5_,^8^ and (c) resistance of Fe_4_O_5_ at 298 K upon compression and decompression. A part of the *R*(*P*) plot is expanded in the inset to emphasize the change of the *R*(*P*) behavior at 88 GPa.

**Figure 3:**
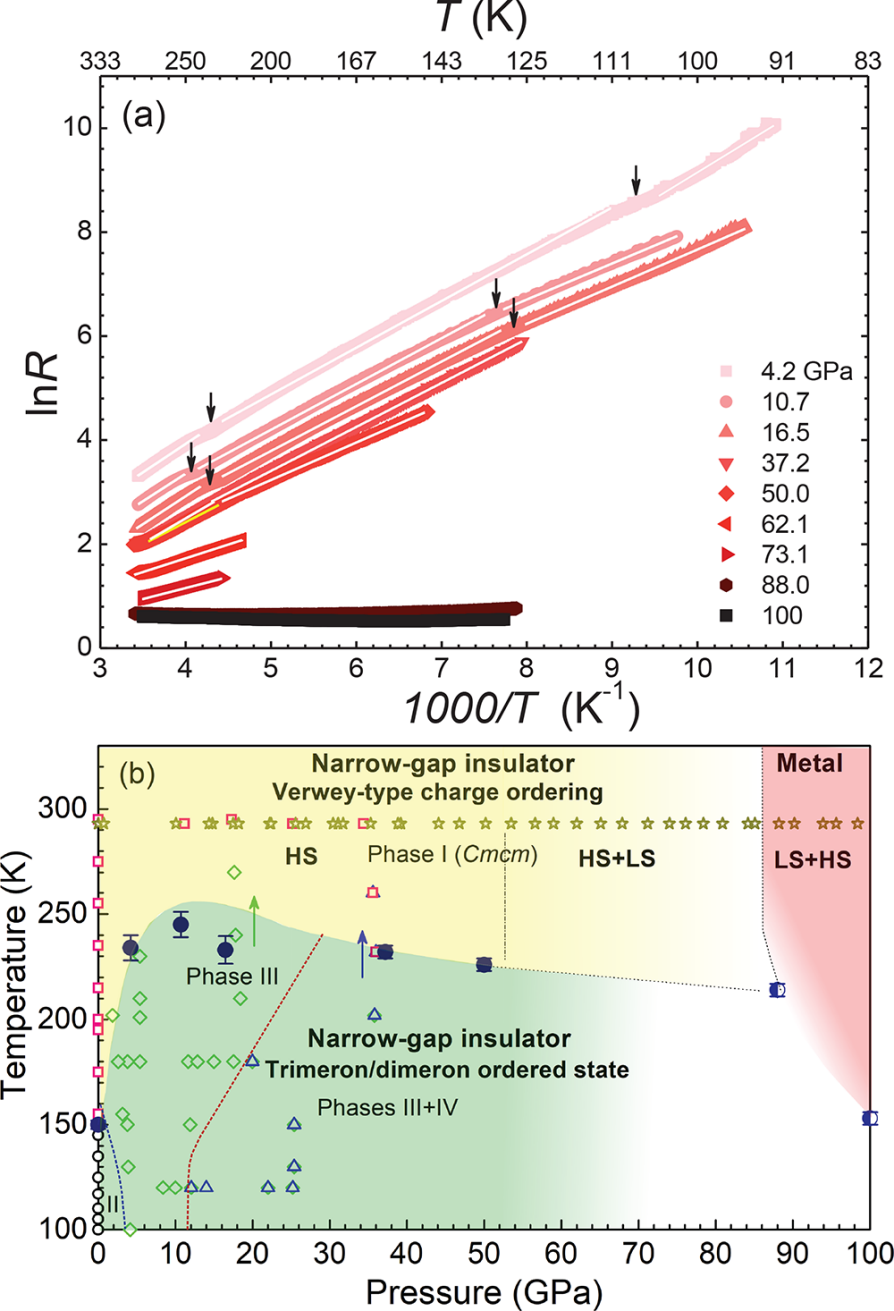
(a) Temperature-dependent resistance measured for different pressures plotted as ln(*R*) vs 1/*T*. Note the slope changes at about 240 and 110–130 K (arrows indicate kinks) coinciding with the onset of novel charge-ordered phases.^8^ (b) Pressure–temperature phase diagram of charge ordered states of Fe_4_O_5_. The phase diagram is based on the resistance measurements, namely, the *P*, *T* points (solid circle symbols) corresponding with the change of electrical transport activation energy (see Figure 3a) and results of single crystal XRD experiments (present and recently published^8^). Experimental points on the diagram corresponding to different structural phases are shown in different open symbols: stars, squares, Phase-I (*Cmcm*), present and measurements from ref (8), respectively; circles, Phase-II;^8^ diamonds, Phase-III;^8^ triangles, Phase-III+IV.^8^ The light-olive-shaded area shows the proposed stability regions of the CO “trimeron”/“dimeron” II, III, and IV phases. The phase boundaries between phases II and III as well as between phases III and III+IV are demarcated by the blue and red dashed lines, respectively.^8^ The yellow-shaded area shows the proposed stability regions of the Verwey-type CO Phase-I. The dotted line, separated Verwey-type and “trimeron”/“dimeron” CO states above 50 GPa, is only a guide for eyes drawn as an approximation of the low-pressure data and our *R*(*P*, *T*) measurements above 50 GPa. The vertical dashed-dotted and dashed lines demarcate the pressures where electronic transitions are onset (LP → HP1 → HP2, respectively, see text). We note that the spin state of HP1 phase changes gradually with a pressure increase due to the increase of LS abundance. The bottom edge of the metal region based on the *P*, *T* points (half-solid circles) corresponding with the change of *R*(*T*) slope sign. The arrows show the directions of the temperature variation during the experiments.^8^ Some points show the phase coexistence. We note that according to ref (8), since the basic structural reflections of the Phase-I and Phase-III structures are identical, the region of their coexistence could not be properly delineated.

In order to clarify the electronic state of Fe_4_O_5_, we perform electrical resistance measurements. Our findings are summarized in Figures 2 and 3. Up to about 88 GPa, our results show an activation type behavior of the temperature-dependent resistance *R*(*T*) below 298 K. Our estimate of the activation energy (at 240–290 K temperature range) shows its weak dependence upon moderate compression to ∼50 GPa, *E*_a_ ∼80 meV (see Figure 2b). This implies that below 50 GPa, Fe_4_O_5_ is a narrow gap semiconductor with an energy gap of ∼0.16 eV, compatible with that of magnetite below *T*_V_, ∼0.14 eV.^48,49^ Upon further compression, *E*_a_ gradually decreases and at ∼88 GPa *R*(*T*) shows a *metallic* behavior. We note, however, differences in the onset pressures of the IMT as discerned by *R*(*P*, *T*) (∼88 GPa) and XRD (∼84 GPa) measurements. In principle, this may be attributed to the different pressure transmitting media used and how the degree of nonhydrostaticity affects the electronic transition. In addition, in the synchrotron XRD measurements, the signal derives from a small central part of the sample, whereas in electrical transport studies the signal is collected from a much larger region of the sample diameter, resulting in possible pressure gradient effects which could be significant in determining phase transition pressures. Thereby, we conclude that at ∼88 GPa, RT Fe_4_O_5_ undergoes metallization, which is consistent with an appreciable increase of its bulk modulus. This implies that at pressures below 88 GPa (i.e., in the insulating phase), RT Fe_4_O_5_ exhibits localization of the Fe 3d state, which taking into account the mixed-valence nature of Fe_4_O_5_ presumes its charge ordering (see below). Moreover, we note that at ∼88 GPa the slope of *R*(*T*) curves changes from positive to negative with a temperature decrease (Figure 2a, inset) suggesting a carrier localization at low temperatures.

Our results reveal an appreciable decrease of the pressure-dependent resistance *R*(*P*) upon compression to ∼25 GPa at RT and then an additional drop above ∼50 GPa, while metallization at ∼88 GPa is accompanied by an appreciable change of the *R*(*P*) slope (see the inset of Figure 2c). Moreover, upon decompression, *R*(*P*) does not exhibit any visible hysteresis. Under pressure, we notice an anomalous variation of the inverse-temperature dependence of ln(*R*(1/*T*)) at about 230–245 and 110–130 K (see Figure 3a). We propose that these anomalies in *R*(1/*T*) behavior are associated with different CO transitions observed in Fe_4_O_5_ at low temperature below 40 GPa.^8^ As was previously shown, the latter are accompanied by a complex structural rearrangement associated with the formation of iron trimerons and dimerons (i.e., with the formation of charge density wave with Fe^(2.5±δ)+^ and δ < 0.5 in the structurally distinct Fe1, Fe2, and Fe3 sites).^7,8^

### 3.3 DFT+DMFT Results: Verwey-Type CO at Low Pressures

**Figure 4:**
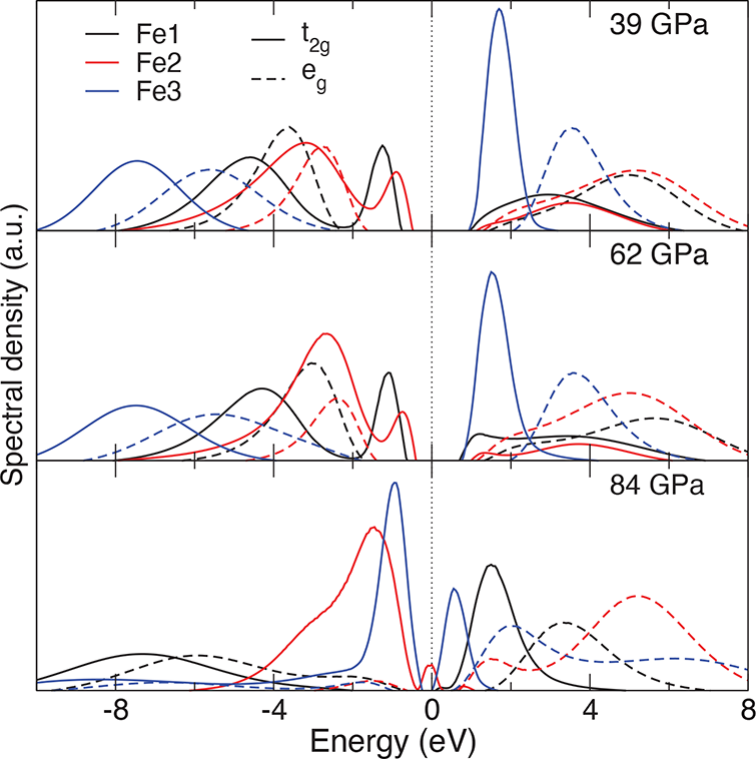
Fe *t*_2*g*_ and *e*_*g*_ spectral functions of PM Fe_4_O_5_ as obtained by DFT+DMFT for different pressures. We note that all three crystallographically distinct Fe1, Fe2, and Fe3 sites are seen to be Mott insulating at 39 and 62 GPa (for *U* = 6 eV). At ∼84 GPa, the prismatic Fe1 sites remain to be Mott insulating, while we observe metallization of the octahedral Fe2 and Fe3 sites (due to the Fe *t*_2*g*_ states).^52^

Next, we perform a microscopic calculation of the electronic structure and magnetic state of Fe_4_O_5_ using a fully charge self-consistent DFT+DMFT method.^36,37^ Our DFT+DMFT results for the Fe 3d spectral functions of Fe_4_O_5_ are summarized in Figure 4. We find that paramagnetic Fe_4_O_5_ (at 39 GPa) is a correlated (Mott) insulator characterized by charge ordering of Fe 2+ (“ferrous”) and 3+ (“ferric”) cations at the structurally distinct Fe1/Fe2 and the Fe3 sites, respectively, with a relatively large d–d energy gap of ∼1.3 eV. We find that at 39 GPa, the Fe 3d electrons are strongly localized and form the HS state for all the Fe sites. The local magnetic moments are 3.97, 3.21, and 4.85 μ_B_ for the prismatic Fe1 and the octahedral Fe2 and Fe3 sites, respectively (see Table 1). The corresponding fluctuating moments *M*_loc_ are 3.94, 3.05, and 4.32 μ_B_.^50^ The energy gap sits between the occupied Fe1 and Fe2 *t*_2*g*_ and unoccupied Fe *t*_2*g*_ states. Interestingly that the energy difference between the Fe3 3d states is large of about 4 eV, while for the Fe1 and Fe2 3d states is of ∼2 eV. It seems to relate to a different electronic (valence) state of these sites and, hence, to a different degree of correlation strength of the Fe 3d electrons. We note that the energy gap depends very sensitively on the choice of the Hubbard *U* value of the multiorbital Hubbard model solved within DFT+DMFT, reducing to ∼0.3 eV for *U* = 5 eV,^51^ compatible with that in the experiment.

**Table 1:** Fe 3d Local Magnetic Moments (*M*), Wannier Fe 3d Electron Occupations (*N*_d_), the HS/LS/IS Spin-State Weights (*W*_s_) of Fe Ions and the Weights of the Fe 3d^5^, d^6^, and d^7^ Atomic Configurations (*W*_d_) of Fe_4_O_5_ Evaluated by DFT+DMFT with *U* = 6 eV and *J* = 0.89 eV for Different Pressures

Fe ion		39 GPa	62 GPa	84 GPa
Fe1	*M*, μ_B_	3.97	3.97	4.85
Fe2		3.21	2.66	1.7
Fe3		4.85	4.7	3.1
				
Fe1	*N*_d_	5.97	5.93	5.08
Fe2		5.98	5.96	5.93
Fe3		5.03	5.05	5.55
				
	*W*_s_		HS/LS/IS	
Fe1		0.96 0.00 0.04	0.94 0.01 0.05	0.98 0.00 0.02
Fe2		0.64 0.33 0.03	0.43 0.55 0.02	0.14 0.84 0.02
Fe3		0.96 0.03 0.01	0.90 0.09 0.01	0.38 0.60 0.02
				
	*W*_d_		d^5^ d^6^ d^7^	
Fe1		0.04 0.94 0.02	0.08 0.90 0.02	0.86 0.11 0.00
Fe2		0.04 0.94 0.02	0.05 0.92 0.02	0.09 0.88 0.03
Fe3		0.94 0.05 0.00	0.89 0.08 0.00	0.42 0.55 0.02

Our analysis of the Fe 3d Wannier occupations and local moments are consistent with robust charge ordering of Fe^2+^/Fe^3+^ cations (at low pressures). In particular, we observe a substantial charge disproportionation with the Wannier Fe1, Fe2, and Fe3 3d orbital occupations of 5.97, 5.98, and 5.03, respectively, implying a 2+ valence state for the Fe1 and Fe2 and 3+ for the Fe3 sites. On the other hand, our results for the electron occupations obtained by integration of charge density inside the atomic sphere with radius 0.86 Å are only 5.31, 5.34, and 5.18 for the Fe1, Fe2, and Fe3 sites, respectively. That is, the physical charge density difference around the structurally distinct prismatic Fe1 and octahedral Fe2 and Fe3 sites is weak, ∼0.14. This implies the importance of Fe 3d to O 2p charge transfer, i.e., the charge disproportionation is significantly screened due to the Fe 3d–O 2p charge redistribution, with Fe^(2.5±δ)+^ and δ ≪ 0.5.^21,25^ Our results for charge disproportionation agree well with previous estimates for the low-temperature charge-ordered phases of the mixed-valence oxides such as Fe_3_O_4_ and rare-earth nickelates RNiO_3_, which give a 20–40% of the ideal ionic disproportionation.^21−23,25,29,42,53−55^

In addition, our results for the decomposition of the electronic state into atomic configurations (valence states) give direct evidence for charge ordering. We find that the valence value for the prismatic Fe1 and octahedral Fe2 sites is nearly 2+ (see Table 1; the Wannier 3d^6^ configuration has a weight of about 94% with a 2% admixture of the 3d^5^ state), while the Fe3 sites are 3+ . Most interestingly, this valence configuration is consistent with the Verwey-type Fe^2+^/Fe^3+^ CO model.^19^ In fact, assuming that the Fe1 and Fe2 sites adopt a 2+ state, while the Fe3 sites are 3+, we obtain the Verwey-type CO model,^19,20^ with a simple charge arrangement of iron (010) planes of Fe_4_O_5_ alternately occupied by 2+ and 3+ Fe cations. Our results for the decomposition of electronic state into atomic spin-state configurations within DFT+DMFT show that all the Fe sites are in the HS state (see Table 1).^36,56^

### 3.4 Site-Selective Mott IMT at High Pressures

Our DFT+DMFT results further demonstrate a series of complicated pressure-induced electronic and magnetic state transformations in PM Fe_4_O_5_. At 62 GPa, it is a correlated insulator with an energy gap of ∼0.95 eV characterized by a site-selective collapse of local moments. The calculated local magnetic moments are ∼3.97, 2.66, and 4.7 μ_B_ for the prismatic Fe1 and the octahedral Fe2 and Fe3 sites, respectively, implying a significant reduction by ∼17% of the octahedral Fe2 moments under pressure. We conclude that above ∼50 GPa the Fe2 magnetic moments undergo a HS–LS state crossover, while the Fe1 and Fe3 sites remain in the HS state. In accordance with this, our analysis of atomic spin-state configurations shows that for the Fe2 sites, the LS state has a predominant weight of ∼55% with a large ∼43% admixture of the HS state (the latter is due to quantum mixing and temperature effects). Moreover, for the Hubbard *U* = 5 eV, we obtain the site-selective Mott insulator phase with the Fe2 3d states being a LS metal, while the Fe1 and Fe3 states are insulating (with localized 3d electrons). Taking into account that our resistance measurements reveal an activation-type behavior below ∼88 GPa, we conclude that at about 50 GPa Fe_4_O_5_ adopts the site-selective Mott phase. The latter is characterized by site-dependent collapse of local moments at the octahedral Fe2 2+ sites, in close similarity to the behavior of the insulating phases of rare-earth nickelates, RNiO_3_ (although the microscopic origins of CO in RNiO_3_ and Fe_4_O_5_ might be significantly different).^53−55^

The HS–LS crossover at the Fe2 sites is accompanied by redistribution of charges between the *t*_2*g*_ and *e*_*g*_ orbitals within the Fe2 3d shell. Fe2 *t*_2*g*_ orbital occupations are found to increase with pressure, resulting in a (nearly) fully occupied state at 62 GPa (*t*_2*g*_ occupation is ∼0.82, while *e*_*g*_ is 0.26). We note that this phase transition does not affect charge disproportionation between the Fe sites. The Fe 3d Wannier orbital occupancies are close to that at 39 GPa (5.93, 5.96, and 5.05, respectively), implying the robust Verwey-type CO. This agrees well with an activated behavior of electrical resistivity up to about 86 GPa. In accordance with this, the valence value for the prismatic Fe1 and octahedral Fe2 sites are nearly 2+, while the Fe3 sites are 3+.

Upon further compression to ∼84 GPa, we obtain the site-selective Mott phase characterized by a site-dependent metallization of the 3d electrons. In DFT+DMFT, we found significantly reduced local magnetic moments for the octahedral Fe2 and Fe3 site, ∼1.7 and 3.1 μ_B_, whereas for the Fe1 sites the local moments increase to 4.85 μ_B_. We note that the pressure-induced increase of the local moments at the prismatic Fe1 sites, from 3.97 to 4.85 μ_B_, is highly unexpected, and as we will show, it is related to a change of the valence state of the Fe1 ions.

While the octahedral Fe2 and Fe3 3d electrons are metallic and seems to be predominantly in the LS state (see Table 1), the prismatic Fe1 3d states remain in the HS state and are Mott insulating (see Figure 4; the Fe3 3d states are nearly metallic, becoming a good metal for *U* = 5 eV). Most notably, the phase transition is accompanied by a change of the valence state of the prismatic Fe1 sites from 2+ to 3+ (its Fe 3d Wannier occupation is now ∼5.08, with the 3d^5^ state weight of 86%), while the octahedral Fe sites are mixed-valent Fe 2+/3+. This suggests that above ∼84 GPa, Fe_4_O_5_ undergoes a site-selective Mott IMT which is accompanied by a Fe 2+ to 3+ valence crossover at the Fe1 sites (with an alignment of the valence state of the Fe2 and Fe3 sites). Note that the site-selective Mott insulator phase of Fe_4_O_5_ with site-dependent metallization closely resembles that recently found in HP Fe_2_O_3_.^16,17^ Indeed, our calculations suggest that above 84 GPa, the prismatic Fe1 sites undergo a valence crossover but still remain insulating, while the octahedral Fe2 and Fe3 sites are mixed-valent metallic. This seems to be related with a collapse of the Verwey-type CO and agrees well with a suppression of activation energy and metallic behavior observed above ∼88 GPa in our resistance measurements as well as with a change of compressional behavior of Fe_4_O_5_ found in our XRD.

Interestingly, more simplified spin-polarized DFT electronic structure calculations of the compressional behavior of Fe_4_O_5_ with a long-range antiferromagnetic ordering also predict two consecutive pressure-induced HS-LS crossovers at the octahedral Fe sites, first at Fe2 at ∼58 GPa and then at Fe3 above 80 GPa, while the prismatic Fe1 ions remain in the HS state up to a high pressure of ∼150 GPa (see the Supporting Information). Moreover, our DFT calculations show a sizable decrease of the bulk modulus (i.e., softening of the lattice) associated with the HS-LS crossover at the octahedral Fe2 sites, from *K*_0_ = 172 GPa in the LP phase to 140 GPa in the HP1 phase, consistent with our experimental and DFT+DMFT results. The calculated phase transition at ∼58 GPa is accompanied by a volume collapse of ∼3.5%, while above ∼80 GPa we found a HS–LS crossover at the octahedral Fe3 sites accompanied by a reduction of the unit-cell volume by ∼1.5% and by a bulk modulus increase to ∼176 GPa (in the HP2 phase). We note however that the DFT calculations cannot explain the insulating state of Fe_4_O_5_, yielding a metallic solution. Moreover, they cannot properly explain a 2+ to 3+ valence crossover at the prismatic Fe sites at high pressure which, according to our DFT+DMFT results, is related to the site-selective Mott IMT (metallization of the octahedral Fe2 and Fe3 sites, while the prismatic Fe1 sites remain Mott localized). This implies the crucial importance of the effects of strong electronic correlations, consistent with previous studies of the Mott IMT in correlated oxides.^16,17,53^

## 4 Discussion

For Fe_4_O_5_, our DFT+DMFT calculations show a significant increase of the abundance of the LS state at the octahedral Fe sites with a pressure increase to 62 GPa and then to 84 GPa. The calculated onset of the HS–LS crossover at ∼50 GPa is in excellent agreement with the observed sharp volume drop of ∼2.4% and shrinkage of the *b* axis and Fe–O octahedral distances at this pressure range. It is noteworthy that a very similar volume drop associated with a spin-state crossover of the octahedral Fe2 ions at ∼58 GPa was also obtained by the more simplified spin-polarized DFT calculations. Thus, we conclude that the experimentally observed phase transition at ∼50 GPa is driven by a HS–LS crossover of the octahedral Fe2 sites.

Moreover, the gradual nature of the spin transition (at finite temperatures), suggested by the DFT+DMFT calculations, elucidates a drastic change in the compressional behavior of Fe_4_O_5_ above 56 GPa. Such a gradual spin-state transition should be accompanied by a sluggish decrease in the unit-cell volume that explain the apparent softness of HP Fe_4_O_5_, which contradicts with the anticipated hardening of the lattice at the spin-state transition or Mott IMT.^13,16,18,57−60^ Thus, the experimentally observed behavior of the crystal volume is attributed to the interplay between normal compressibility and spin variation effect on the Fe ionic radius.^60^ This is in agreement with the DFT calculations showing a sizable decrease of the bulk modulus associated with the HS–LS crossover at the octahedral Fe2 sites. Most notably, an estimate of the relative difference in polyhedral volumes, expected for the HS and LS FeO_6_ octahedra for Fe^2+^ and Fe^3+^ ions, gives ∼21% and 12%, respectively,^57,58^ which significantly exceeds the volume change observed at ∼50 GPa and an ∼5.2% reduction of the volume at ∼100 GPa (as compared to the equation of states for the LP phase, see Figure 1a). These estimations are in qualitative agreement with our theoretical calculations showing a progressive site-selective collapse of local magnetic moments at the octahedral Fe sites, not completed at ∼84 GPa, and metallization.

We note that according to our *R*(*P*, *T*) measurements, the electrical transport activation energy (charge gap) gradually decreases above ∼50 GPa, corroborating with the onset of the HS–LS crossover at the Fe2 sites. This suggests that the HS–LS transition under pressure affects the charge gap caused by the Verwey-type CO and finally leads to its suppression. In fact, our electrical transport and XRD measurements show that at ∼88 GPa, RT Fe_4_O_5_ undergoes a reversible insulator-to-metal phase transition, which coincides with a subtle volume drop and appreciable bulk modulus increase. This implies a collapse of the Verwey-type CO under pressure above 88 GPa and corresponds well with the DFT+DMFT results, which show that the site-dependent spin-state crossover on the octahedral Fe sites of Fe_4_O_5_ is followed by a site-selective Mott IMT above ∼84 GPa. According to our DFT+DMFT calculations, this phase transition is accompanied by a drastic change of the valence state of the Fe sites suggesting that the site-selective electron delocalization above ∼84 GPa concurs with a substantial redistribution of the charge density between the different Fe sites. This anomalous high-pressure behavior of Fe_4_O_5_ corroborates with a highly unusual softening of the lattice at ∼50 GPa that in accordance with XRD the bulk modulus drops from *K*_0_ ∼190 to 102 GPa in the HP1 phase, and upon further compression above 84 GPa, *K*_0_ increases to ∼140 GPa in the HP2 phase.

Most notably, our DFT+DMFT calculations show that the Mott insulating state of RT Fe_4_O_5_ below ∼84 GPa is accompanied by the Verwey-type CO of the crystallographically distinct Fe sites. In fact, our theoretical calculations (consistent with the BVS analysis) show that the prismatic Fe1 and octahedral Fe2 sites adopt a 2+ state, while the octahedral Fe3 sites are 3+. This leads to a simple charge arrangement of iron (010) planes of Fe_4_O_5_ alternately occupied by 2+ and 3+ Fe cations, reminiscent of that of the CO proposed by Verwey in Fe_3_O_4_.^19^ With this, the physical charge density difference around the structurally distinct prismatic Fe1 and octahedral Fe2 and Fe3 sites (calculated taking into account a significant screening due to the Fe 3d–O 2p charge redistribution, with Fe^(2.5±δ)+^ and δ < 0.5) is weak, ∼0.14, consistent with our BVS analysis. The latter value agrees well with previous estimates for charge disproportionation in the low-temperature charge-ordered phases of the mixed-valence oxides such as Fe_3_O_4_ and rare-earth nickelates RNiO_3_, which give 20–40% of the nominal ionic disproportionation.^21−23,25,29,42,53−55^ Meanwhile, our calculations reveal a drastic change of the valence state of the Fe sites above ∼84 GPa, suggesting that metallization of RT Fe_4_O_5_ concurs with a collapse of CO. This implies that the Mott insulating state of Fe_4_O_5_ below ∼84 GPa is driven by the complex interplay of electronic correlation (Mott localization) and CO effects (localization due to electron–lattice coupling and long-range electron–electron repulsion).

Moreover, our analysis suggests that Fe_4_O_5_ provides a “missing link” in understanding of the Verwey charge-ordering phenomenon in mixed-valence materials, showing a transition from the narrow-gap insulator with the Verwey-type CO at RT to a complicated “trimeron” and “dimeron” ordered state at low temperatures.^7,8^ Summing up the available data, we present in Figure 3b the pressure–temperature phase diagram of charge ordered states of Fe_4_O_5_ based on our resistivity and XRD results combined with the previous XRD results from ref (8).

Based on our results, we propose the following microscopic model which explains the complex electronic and charge ordering behavior of Fe_4_O_5_ under pressure. In fact, although the Verwey CO model possesses the minimum electrostatic repulsion energy (e.g., in Fe_3_O_4_), the “trimeron” and “dimeron” ordering is stabilized due to a complex competition between the electrostatic and elastic contributions in the total energy.^25^ It leads to a “partial” occupation of the Fe (001) planes that makes the difference between the [010] and the [001]/[100] directions less pronounced. This reduces the lattice stress and, as a result, reduces the “elastic” energy. We propose that this competition is the primary cause for development of the complex CO in low-temperature Fe_4_O_5_. We believe that the same mechanism is responsible for CO in the low-temperature phases of mixed-valent *n*FeO·*m*Fe_2_O_3_^13^ (e.g., Fe_5_O_6_ shows a very similar *R*(*P*, *T*) behavior^11^). In contrast to RT Fe_3_O_4_ with mixed-valence Fe^2+^/Fe^3+^ ions occupying identical octahedral positions of the cubic structure, in Fe_4_O_5_, localization of the Fe 3d charges occurs on the structurally distinct prismatic Fe1 and octahedral Fe2 and Fe3 sites, which makes charge disproportionation more feasible. It is also interesting that upon a moderate compression of ∼25 GPa, Fe_3_O_4_ undergoes a structural phase transition (with a symmetry change), while in Fe_4_O_5_ the *Cmcm* crystal phase (CaFe_3_O_5_-phase) persists up to ∼100 GPa. This seems to explain the anomalous stability of the Verwey-type CO in RT Fe_4_O_5_ until high pressure ∼84 GPa.

We also note that a complexity of the Fe_4_O_5_ compound results in much more intricate phase diagram compared to the case of magnetite (see refs (24) and (61)). Furthermore, an onset of metallization in Fe_4_O_5_ takes place at pressures above 86 GPa at RT and above 1 Mbar at the low-temperature range, while in Fe_3_O_4_ a charge-ordered insulating phase comprises a relatively small pressure–temperature range (below 8 GPa and 120 K). While the details of the high pressure behavior of Fe_4_O_5_ are still unsettled, our results indicate the existence of a triple point near ∼88 GPa and 220 K in the phase diagram of Fe_4_O_5_ (see Figure 3b). Most notably, the compressional behavior of Fe_4_O_5_ “unifies” the behavior of both the rare-earth nickelates RNiO_3_ and highly pressurized Fe_2_O_3_ at moderate and high pressures, respectively, showing a site-selective collapse of magnetic moments in the Mott insulating phase at moderate pressures and site-dependent metallization of the Fe 3d electrons under high pressures.^16,17,53,55^

## 5 Conclusions

In conclusion, using single-crystal and powder XRD, in combination with resistance measurements and the DFT+DMFT electronic structure calculations, we show that at low pressures RT Fe_4_O_5_ is a narrow-gap correlated insulator with an orthorhombic CaFe_3_O_5_-type crystal structure characterized by the “classical” Verwey-type charge ordering of Fe 2+ and 3+ ions. We found that Fe_4_O_5_ undergoes a series of complicated pressure-induced electronic and magnetic state transformations characterized by a site-selective collapse of local moments and metallization. In particular, we found a HS–LS state crossover to set in for a fraction of the octahedral Fe^2+^ ions starting at ∼50 GPa, which results at higher pressures in the site-selective Mott insulator with metallization of the octahedral Fe states. Upon compression above ∼84 GPa, our DFT+DMFT calculations show a Fe 2+ to 3+ valence crossover at the prismatic Fe1 sites, accompanied by a mixed-valence metallic behavior of the octahedral Fe sites, consistent with our resistance measurements. The phase transitions are electronic in origin and are associated with the site-selective Mott IMT. It leads to a drastic change of compressional behavior of Fe_4_O_5_ at high pressures. Most notably, the Verwey-type CO is found to compete with the “trimeron”/“dimeron” charge ordered states at low temperatures, allowing for a pressure and temperature tuning of CO in the system. This behavior explains a rich diversity of different types of CO patterns experimentally observed in Fe_4_O_5_ under pressure.

## Supporting Information Available

Technical details about the methods used, detailed discussion of the crystal structure properties of Fe_4_O_5_ under pressure, and additional characterization data and spectra (PDF)

## References

[ref1] Mott N. F. Metal-Insulator Transitions; Taylor & Francis: London, 1990.

[ref2] Imada M.; Fujimori A.; Tokura Y. Metal-insulator transitions. Rev. Mod. Phys. 1998, 70, 1039 10.1103/RevModPhys.70.1039.

[ref3] Tokura Y.; Nagaosa N. Orbital physics in transition-metal oxides. Science 2000, 288, 462 10.1126/science.288.5465.462. 10775098

[ref4] Dagotto E. Complexity in strongly correlated electronic systems. Science 2005, 309, 257 10.1126/science.1107559. 16002608

[ref5] Lavina B.; Dera P.; Kim E.; Meng Y.; Downs R. T.; Weck P. F.; Sutton S. R.; Zhao Y. Discovery of the recoverable high-pressure iron oxide Fe_4_O_5_. Proc. Natl. Acad. Sci. U.S.A. 2011, 108, 17281 10.1073/pnas.1107573108. 21969537 PMC3198347

[ref6] Woodland A. B.; Frost D. J.; Trots D. M.; Klimm K.; Mezouar M. In situ observation of the breakdown of magnetite (Fe_3_O_4_) to Fe_4_O_5_ and hematite at high pressures and temperatures. Am. Mineral. 2012, 97, 1808 10.2138/am.2012.4270.

[ref7] Ovsyannikov S. V.; Bykov M.; Bykova E.; Kozlenko D. P.; Tsirlin A. A.; Karkin A. E.; Shchennikov V. V.; Kichanov S. E.; Gou H.; Abakumov A. M.; Egoavil R.; Verbeeck J.; McCammon C.; Dyadkin V.; Chernyshov D.; van Smaalen S.; Dubrovinsky L. S. Charge-ordering transition in iron oxide Fe_4_O_5_ involving competing dimer and trimer formation. Nature Chem. 2016, 8, 501 10.1038/nchem.2478. 27102685

[ref8] Ovsyannikov S. V.; Bykov M.; Bykova E.; Glazyrin K.; Manna R. S.; Tsirlin A. A.; Cerantola V.; Kupenko I.; Kurnosov A. V.; Kantor I.; Pakhomova A. S.; Chuvashova I.; Chumakov A. I.; Rüffer R.; McCammon C.; Dubrovinsky L. S. Pressure tuning of charge ordering in iron oxide. Nat. Commun. 2018, 9, 4142 10.1038/s41467-018-06457-x. 30297769 PMC6175922

[ref9] Lavina B.; Meng Y. Unraveling the complexity of iron oxides at high pressure and temperature: Synthesis of Fe_5_O_6_. Sci. Adv. 2015, 1, e1400260 10.1126/sciadv.1400260. 26601196 PMC4640612

[ref10] Hikosaka K.; Sinmyo R.; Hirose K.; Ishii T.; Ohishi Y. The stability of Fe_5_O_6_ and Fe_4_O_5_ at high pressure and temperature. Am. Mineral. 2019, 104, 1356 10.2138/am-2019-7097.

[ref11] Ovsyannikov S. V.; Bykov M.; Medvedev S. A.; Naumov P. G.; Jesche A.; Tsirlin A. A.; Bykova E.; Chuvashova I.; Karkin A. E.; Dyadkin V.; Chernyshov D.; Dubrovinsky L. S. A room-temperature Verwey-type transition in iron oxide, Fe_5_O_6_. Angew. Chem., Int. Ed. 2020, 59, 5632 10.1002/anie.201914988. PMC7154779 31899577

[ref12] Sinmyo R.; Bykova E.; Ovsyannikov S. V.; McCammon C.; Kupenko I.; Ismailova L.; Dubrovinsky L. Discovery of Fe_7_O_9_: a new iron oxide with a complex monoclinic structure. Sci. Rep. 2016, 6, 32852 10.1038/srep32852. 27605075 PMC5015080

[ref13] Bykova E.; Dubrovinsky L.; Dubrovinskaia N.; Bykov M.; McCammon C.; Ovsyannikov S. V.; Liermann H.-P.; Kupenko I.; Chumakov A.; Rüffer R.; Hanfland M.; Prakapenka V. Structural complexity of simple Fe_2_O_3_ at high pressures and temperatures. Nat. Commun. 2016, 7, 10661 10.1038/ncomms10661. 26864300 PMC4753252

[ref14] Hu Q.; Kim D. Y.; Yang W.; Yang L.; Meng Y.; Zhang L.; Mao H. K. FeO_2_ and FeOOH under deep lower-mantle conditions and Earth’s oxygen-hydrogen cycles. Nature (London) 2016, 534, 241 10.1038/nature18018. 27279220

[ref15] Streltsov S. S.; Shorikov A. O.; Skornyakov S. L.; Poteryaev A. I.; Khomskii D. I. Unexpected 3+ valence of iron in FeO_2_, a geologically important material lying in between oxides and peroxides. Sci. Rep. 2017, 7, 13005 10.1038/s41598-017-13312-4. 29021556 PMC5636914

[ref16] Greenberg E.; Leonov I.; Layek S.; Konopkova Z.; Pasternak M. P.; Dubrovinsky L.; Jeanloz R.; Abrikosov I. A.; Rozenberg G. Kh. Pressure-induced site-selective Mott insulator-metal transition in Fe_2_O_3_. Phys. Rev. X. 2018, 8, 031059 10.1103/PhysRevX.8.031059.

[ref17] Leonov I.; Rozenberg G. Kh.; Abrikosov I. A. Charge disproportionation and site-selective local magnetic moments in the post-perovskite-type Fe_2_O_3_ under ultra-high pressures. npj Comput. Mater. 2019, 5, 90 10.1038/s41524-019-0225-9.

[ref18] Greenberg E.; Xu W. M.; Nikolaevsky M.; Bykova E.; Garbarino G.; Glazyrin K.; Merkel D. G.; Dubrovinsky L.; Pasternak M. P.; Rozenberg G. Kh. High-pressure magnetic, electronic and structural properties of *M*Fe_2_O_4_ (*M* = Mg, Zn, Fe) ferric spinels. Phys. Rev. B 2017, 95, 195150 10.1103/PhysRevB.95.195150.

[ref19] Verwey E. J. W. Electronic conduction of magnetite (Fe_3_O_4_) and its transition point at low temperatures. Nature (London) 1939, 144, 327 10.1038/144327b0.

[ref20] Walz F. The Verwey transition - a topical review. J. Phys.: Condens. Matter 2002, 14, R285 10.1088/0953-8984/14/12/203.

[ref21] Wright J. P.; Attfield J. P.; Radaelli P. G. Long Range Charge Ordering in Magnetite Below the Verwey Transition. Phys. Rev. Lett. 2001, 87, 266401 10.1103/PhysRevLett.87.266401. 11800847

[ref22] Senn M. S.; Wright J. P.; Attfield J. P. Charge order and three-site distortions in the Verwey structure of magnetite. Nature (London) 2012, 481, 173 10.1038/nature10704. 22190035

[ref23] Perversi G.; Pachoud E.; Cumby J.; Hudspeth J. M.; Wright J. P.; Kimber S. A. J.; Attfield J. P. Co-emergence of magnetic order and structural fluctuations in magnetite. Nat. Commun. 2019, 10, 2857 10.1038/s41467-019-10949-9. 31253806 PMC6599026

[ref24] Rozenberg G. Kh.; Hearne G. R.; Pasternak M. P.; Metcalf P. A.; Honig J. M. Nature of the Verwey transition in magnetite (Fe_3_O_4_) to pressures of 16 GPa. Phys. Rev. B 1996, 53, 6482 10.1103/PhysRevB.53.6482. 9982047

[ref25] Leonov I.; Yaresko A. N.; Antonov V. N.; Korotin M. A.; Anisimov V. I. Charge and Orbital Order in Fe_3_O_4_. Phys. Rev. Lett. 2004, 93, 146404 10.1103/PhysRevLett.93.146404. 15524820

[ref26] Piekarz P.; Parlinski K.; Oleś A. M. Mechanism of the Verwey transition in magnetite. Phys. Rev. Lett. 2006, 97, 156402 10.1103/PhysRevLett.97.156402. 17155347

[ref27] Nazarenko E.; Lorenzo J. E.; Joly Y.; Hodeau J. L.; Mannix D.; Marin C. Resonant X-ray diffraction studies on the charge ordering in magnetite. Phys. Rev. Lett. 2006, 97, 056403 10.1103/PhysRevLett.97.056403. 17026123

[ref28] Pontius N.; Kachel T.; Schüßler-Langeheine C.; Schlotter W. F.; Beye M.; Sorgenfrei F.; Chang C. F.; Föhlisch A.; Wurth W.; Metcalf P.; Leonov I.; Yaresko A.; Stojanovic N.; Berglund M.; Guerassimova N.; Düsterer S.; Redlin H.; Dürr H. A. Time-resolved resonant soft x-ray diffraction with free-electron lasers: Femtosecond dynamics across the Verwey transition in magnetite. Appl. Phys. Lett. 2011, 98, 182504 10.1063/1.3584855.

[ref29] Baldini E.; Belvin C. A.; Rodriguez-Vega M.; Ozel I. O.; Legut D.; Kozłowski A.; Oleś A. M.; Parlinski K.; Piekarz P.; Lorenzana J.; Fiete G. A.; Gedik N. Discovery of the soft electronic modes of the trimeron order in magnetite. Nat. Phys. 2020, 16, 541 10.1038/s41567-020-0823-y.

[ref30] Lin J. F.; Wu J.; Zhu J.; Mao Z.; Said A.; Leu B.; Cheng J.; Uwatoko Y.; Jin C.; Zhou J. Sci. Rep. 2015, 4, 06282 10.1038/srep06282. PMC4153994 25186916

[ref31] Chen K.; Baudelet F.; Mijiti Y.; Nataf L.; Di Cicco A.; Hu Z.; Agrestini S.; Komarek A.; Sougrati A. M.; Haines J.; Rouquette J.; Kong Q.; Weng T.-C. J. Phys. Chem. C 2019, 123, 21114 10.1021/acs.jpcc.9b04140.

[ref32] In fact, the low-temperature charge ordered state of Fe_4_O_5_ can be characterized by localized Fe 3d electrons which are distributed over a linear arrangement of two and three neighboring Fe sites and in accordance to ref (22) are called “dimerons” and “trimerons”, respectively. For details see refs (7) and (8).

[ref33] Hong K. H.; Arevalo-Lopez A. M.; Cumby J.; Ritter C.; Attfield J. P. Long range electronic phase separation in CaFe_3_O_5_. Nat. Commun. 2018, 9, 2975 10.1038/s41467-018-05363-6. 30061576 PMC6065443

[ref34] Bindi L.; Shim S.-H.; Sharp T. G.; Xie X. Evidence for the charge disproportionation of iron in extraterrestrial bridgmanite. Sci. Adv. 2020, 6, eaay7893 10.1126/sciadv.aay7893. 31950086 PMC6954055

[ref35] Georges A.; Kotliar G.; Krauth W.; Rozenberg M. Dynamical mean-field theory of strongly correlated fermion systems and the limit of infinite dimensions. Rev. Mod. Phys. 1996, 68, 13 10.1103/RevModPhys.68.13.

[ref36] Leonov I. Metal-insulator transition and local-moment collapse in FeO under pressure. Phys. Rev. B 2015, 92, 085142 10.1103/PhysRevB.92.085142.

[ref37] Pourovskii L. V.; Amadon B.; Biermann S.; Georges A. Self-consistency over the charge density in dynamical mean-field theory: A linear muffin-tin implementation and some physical implications. Phys. Rev. B 2007, 76, 235101 10.1103/PhysRevB.76.235101.

[ref38] Frost D. J.; Poe B. T.; Trønnes R. G.; Liebske C.; Duba A.; Rubie D. C. A new large-volume multianvil system. Phys. Earth Planet. Inter. 2004, 143–144, 507 10.1016/j.pepi.2004.03.003.

[ref39] Anisimov V. I.; Kondakov D. E.; Kozhevnikov A. V.; Nekrasov I. A.; Pchelkina Z. V.; Allen J. W.; Mo S.-K.; Kim H.-D.; Metcalf P.; Suga S.; Sekiyama A.; Keller G.; Leonov I.; Ren X.; Vollhardt D. Full orbital calculation scheme for materials with strongly correlated electrons. Phys. Rev. B 2005, 71, 125119 10.1103/PhysRevB.71.125119.

[ref40] Gull E.; Millis A. J.; Lichtenstein A. I.; Rubtsov A. N.; Troyer M.; Werner P. Continuous-time Monte Carlo methods for quantum impurity models. Rev. Mod. Phys. 2011, 83, 349 10.1103/RevModPhys.83.349.

[ref41] Perdew J. P.; Burke K.; Ernzerhof M. Generalized Gradient Approximation Made Simple. Phys. Rev. Lett. 1996, 77, 3865 10.1103/PhysRevLett.77.3865. 10062328

[ref42] Torrance J. B.; Lacorre P.; Nazzal A. I.; Ansaldo E. J.; Niedermayer Ch. We note that similar, or even smaller, volume anomalies were revealed by precise neutron diffraction studies of the rare-earth nickelates *R*NiO_3_ in the case of temperature-induced insulator-to-metal phase transitions. Phys. Rev. B 1992, 45, 8209 10.1103/PhysRevB.45.8209.

[ref43] Fischer R. A.; Campbell A. J.; Shofner G. A.; Lord O. T.; Dera P.; Prakapenka V. B. Equation of state and phase diagram of FeO. Earth and Planet. Sci. Lett. 2011, 304, 496 10.1016/j.epsl.2011.02.025.

[ref44] Fei Y. Crystal Chemistry of FeO at High Pressure and Temperature, Mineral Spectroscopy: A Tribute to Roger Burns; Dyar M. D., McCammon C., Shaefer M. W., Eds.; Geochemical Society: Houston, TX, 1996.

[ref45] Haavik C.; Stølen S.; Fjellvå g H.; Hanfland M.; Häusermann D. Equation of state of magnetite and its high-pressure modification: Thermodynamics of the Fe-O system at high pressure. Am. Mineral. 2000, 85, 514 10.2138/am-2000-0413.

[ref46] We note that similar results were obtained performing the 3rd order EOS fits to our XRD data (see Table S2). Our results for the LP phase are *V*_0_ = 356.88(3) Å^3^, *K*_0_ = 175(3) GPa, and *K*′ = 5.4(3), which give at 50 GPa *K*_50_ = 417(3) GPa. As expected, this value is higher than the bulk modulus obtained using the 2nd order fit, due to a larger value of *K*′. Fitting the HP1 phase data to the 3rd order EOS results in large errors upon data extrapolation to 0 GPa. Nevertheless, at 50 GPa we obtain *K*_50_ = 293(8) GPa, which is close to that obtained from the 2nd order EOS, ∼279(3) GPa. That is, a significant decrease of bulk modulus at the LP-to-HP1 phase transition is well supported by the data, even taking into account possible errors due to the choice of *K*′.

[ref47] Brown I. D. The Chemical Bond in Inorganic Chemistry: The Bond Valence Model; Oxford University Press, 2002.

[ref48] Park S. K.; Ishikawa T.; Tokura Y. Charge-gap formation upon the Verwey transition in Fe_3_O_4_. Phys. Rev. B 1998, 58, 3717 10.1103/PhysRevB.58.3717.

[ref49] Schrupp D.; Sing M.; Tsunekawa M.; Fujiwara H.; Kasai S.; Sekiyama A.; Suga S.; Muro T.; Brabers V. A. M.; Claessen R. High-energy photoemission on Fe_3_O_4_: Small polaron physics and the Verwey transition. Europhys. Lett. 2005, 70, 789 10.1209/epl/i2005-10045-y.

[ref50] The latter is evaluated as the imaginary-time-average of the local spin susceptibilities χ(τ) = ⟨*m̂*_*z*_(τ)*m̂*_*z*_(0)⟩ as *M*_loc_ = (*k*_B_*T*∫χ(τ) dτ)^1/2^. Here, *m̂*_*z*_(τ) is the instantaneous magnetization on the Ni 3d site at the imaginary time τ, which denotes an imaginary-time evolution ranging from 0 to β = 1/*k*_B_*T* in the path integral formalism.

[ref51] We note that using a smaller Hubbard parameter *U* = 5 eV leads to a shift of the Mott IM transition boundary to smaller pressures without significantly changing the overall picture.

[ref52] To conclude on this, we analyze the behavior of Green function at the imaginary-time contour G(τ = β/2) (β is the inverse temperature, β = 1/*k*_B_*T*), evaluated within DMFT. It gives an estimate of the DOS spectral weight at the Fermi level.

[ref53] Park H.; Millis A. J.; Marianetti C. A. Site-Selective Mott Transition in Rare-Earth-Element Nickelates. Phys. Rev. Lett. 2012, 109, 156402 10.1103/PhysRevLett.109.156402. 23102343

[ref54] Mizokawa T.; Khomskii D. I.; Sawatzky G. A. Spin and charge ordering in self-doped Mott insulators. Phys. Rev. B 2000, 61, 11263 10.1103/PhysRevB.61.11263.

[ref55] Catalano S.; Gibert M.; Fowlie J.; Íñiguez J.; Triscone J.-M.; Kreisel J. Rare-earth nickelates RNiO_3_: thin films and heterostructures. Rep. Prog. Phys. 2018, 81, 046501 10.1088/1361-6633/aaa37a. 29266004

[ref56] Here, the weights of the HS and LS states *W*_s_ are determined as for the HS: and for the LS state: , respectively).

[ref57] Merlini M.; Hanfland M.; Gemmi M.; Huotari S.; Simonelli L.; Strobel P. Fe^3+^ spin transition in CaFe_2_O_4_ at high pressure. Am. Mineral. 2010, 95, 200 10.2138/am.2010.3347.

[ref58] Xu W. M.; Hearne G. R.; Layek S.; Levy D.; Itié J.-P.; Pasternak M. P.; Rozenberg G. Kh.; Greenberg E. Site-specific spin crossover in Fe_2_TiO_4_ post-spinel under high pressure up to nearly a megabar. Phys. Rev. B 2017, 96, 045108 10.1103/PhysRevB.96.045108.

[ref59] Rozenberg G. Kh.; Xu W.; Pasternak M. P. The Mott insulators at extreme conditions; structural consequences of pressure-induced electronic transitions. Zeitschrift für Krist.-Cryst. Mater. 2014, 229, 210–222.

[ref60] We note that a similar behavior was reported for PrFeO_3_, in which a transition of Fe ions to the LS state was not completed with a sharp isostructural phase transition around 50 GPa and continued as a sluggish spin crossover at higher pressures. See, e.g., Rozenberg G. Kh.; Pasternak M. P.; Xu W. M.; Dubrovinsky L. S.; Carlson S.; Taylor R. D. Consequences of pressure-instigated spin crossover in *R*FeO_3_ perovskites; a volume collapse with no symmetry modification. Europhys. Lett. 2005, 71, 228 10.1209/epl/i2005-10071-9.

[ref61] Mori N.; Todo S.; Takeshita N.; Mori T.; Akishige Y. Physica B: Condensed Matter 2002, 312–313, 686 10.1016/S0921-4526(01)01525-3.

