# Supplementary material for: Verwey-Type Charge Ordering and Site-Selective Mott Transition in Fe4O5 under Pressure
Source: J Am Chem Soc. 2022 Jun 1;144(23):10259–69. doi: 10.1021/jacs.2c00895 (PMC9204770; doi:10.1021/jacs.2c00895)
Supplement: Supplementary file 1 — ja2c00895_si_001.pdf [file ja2c00895_si_001.pdf]

Supporting information on "Verwey-type charge ordering and site-selective Mott transition in Fe_4O_5 under pressure"

Samar Layek,[1,2,*] Eran Greenberg,[3,4] Stella Chariton,[3] Maxim Bykov,[5]
Elena Bykova,[6,7] Dmytro M. Trots,[7] Alexander V. Kurnosov,[7] Irina Chuvashova,[8,9]
Sergey V. Ovsyannikov,[7] Ivan Leonov,[10,11,12,†] and Gregory Kh. Rozenberg[1]

[1]*School of Physics and Astronomy, Tel Aviv University, 69978 Tel Aviv, Israel*

[2]*Department of Physics, School of Engineering, University of Petroleum and Energy Studies (UPES), Dehradun, Uttarakhand 248007, India*

[3]*Center for Advanced Radiation Sources, University of Chicago, 5640 South Ellis Ave., 60637 Chicago, USA*

[4]*Applied Physics Division, Soreq NRC, Yavne, 81800, Israel*

[5]*Institute of Inorganic Chemistry, University of Cologne, Greinstrasse 6, 50939 Cologne, Germany*

[6]*Earth and Planets Laboratory, Carnegie Institution for Science, Washington, DC 20015, USA*

[7]*Bayerisches Geoinstitut, Universität Bayreuth, Universitätsstrasse 30, D-95447 Bayreuth, Germany*

[8]*Harvard Physics, Jefferson Physical Lab, 17 Oxford Street, Cambridge, Massachusetts 02138, USA*

[9]*Department of Chemistry and Biochemistry, Florida International University, 11200 SW 8th Street, CP 234, Miami, FL 33199, USA*

[10]*M. N. Miheev Institute of Metal Physics, Russian Academy of Sciences, 620108 Yekaterinburg, Russia*

[11]*Ural Federal University, 620002 Yekaterinburg, Russia*

[12]*Skolkovo Institute of Science and Technology, 143026 Moscow, Russia*

[*]Electronic address: samarlayek@gmail.com
[†]Electronic address: ivan.v.leonov@yandex.ru

Experimental Methods

Samples of Fe_4O_5 were synthesized using a 1200-tonne Multi-Anvil Press at BGI [1] at HP-HT conditions from stoichiometric mixtures of fine powders of Fe_3O_4 and iron (both from Aldrich company, 99.999% purity). These syntheses were performed at pressures of about 14 GPa over several hours. High-quality single crystals with linear sizes of about 20–250 μm were synthesized at high temperatures in the range of 1350–1450 °C [2]. We employed a standard multi-anvil assembly that included a Re cylindrical sample capsule, a $LaCrO_3$ heater, W3Re/W25Re thermocouple, and other components packed inside an octahedron made of 5% Cr_2O_3-doped MgO [3]. The synthesis procedure was similar to the one described in previous works [2, 4]. The chemical composition of the samples was verified in a microprobe analysis using a JEOL JXA-8200 electron microscope. The crystal structure of the samples was determined using single crystal and powder X-ray diffraction (XRD) on a high brilliance Rigaku diffractometer (Mo K_α radiation, $\lambda = 0.7108$ Å).

X-ray diffraction studies

Data collection

Run 1. The data collection was carried out at the ID15B beamline at the European Synchrotron Radiation Facility (ESRF), Grenoble, France (MAR555 flat panel detector, $\lambda = 0.41114$ Å, beam size 10(V) × 10(H) μm^2). Sample-to-detector distance, coordinates of the beam center, tilt angle and tilt plane rotation angle of the detector images were calibrated using Si powder. XRD wide images were collected during continuous rotation of DACs typically from –20° to +20° on ω; while XRD single-crystal data collection experiments were performed by narrow 0.5° scanning of the ±32° ω range. A black single crystal of Fe_4O_5 with dimensions of 0.02 × 0.02 × 0.01 mm^3 together with a small ruby chip (for pressure estimation) were loaded into LeToullec membrane-driven type DAC [5] equipped with Boehler-Almax diamonds with 300 μm culet size. A hole with diameter about 150 μm in steel gasket pre-indented to 40 μm was served as a pressure chamber. Neon was used both as a pressure transmitting medium and as a pressure standard [6].

The DAC was compressed to 46.8(5) GPa with wide and step scan images being collected at each pressure point. Processing of XRD data (the unit cell determination, integration of

the reflection intensities, empirical absorption correction) were performed using CrysAlisPro software [7]. A single crystal of an orthoenstatite [$(Mg_{1.93},Fe_{0.06})(Si_{1.93},Al_{0.06})O_6$, space group *Pbca* with a = 18.2391(3), b = 8.8117(2), and c = 5.18320(10) Å], was used to calibrate the instrument model of CrysAlisPro (sample-to-detector distance, the detector's origin, offsets of the goniometer angles and rotation of the X-ray beam and the detector around the instrument axis).

Run 2. In the second attempt we loaded a mixture of Fe_3O_4 (with purity 99.997% purchased from Alfa Aesar (LOT: 10192900) and Fe (with 99.9+% purity purchased from Alfa Aesar (STOCK: 00170, LOT: J3OK28) powders into a DAC, then compressed the DAC to 13.8 GPa and performed *in-situ* laser-heating for a part of the mixture up to 1820(70) K. This run was carried out at the beamline 13-IDD of the Advanced Photon Source (APS), Argonne National Laboratory (Argonne, IL), with a wavelength of $\lambda = 0.2952$ Å, and a spot-size of $3(\mathrm{V}) \times 4(\mathrm{H})\ \mu\mathrm{m}^2$. Detector tilts and sample to detector distance were calibrated using a LaB_6 standard. The laser heating process formed a few single-crystal grains along with some powder of Fe_4O_5. Then, we compressed these samples up to ∼100 GPa and collected both single-crystal (ω rotation of $-33°$ to $+36°$ with 0.5° steps) and powder diffraction data. Single-crystal diffraction processing was performed in the same manner described in Run 1, and using an orthoenstatite standard from the same batch.

We employed DACs with diamond anvil culet sizes of 300 and 150 μm, respectively. Together with the sample in the same cell we loaded a ruby ball (for the first run) and a chip of gold (for the second run) that was used for pressure determination [6]. The DACs were filled with a Ne pressure-transmitting medium. At each pressure point we acquired the single-crystal X-ray diffraction images upon a continuous rotation of the cell with the sample around the vertical ω-axis with a step of $\Delta\omega = 0.5°$ and an exposure time of $t = 0.5$ s/frame. The diffraction data were collected by a Perkin Elmer XRD1621 detector at DESY and a Pilatus 1M CdTe detector at APS. We analyzed these data with CrysAlisPro software [7] and solved the crystal structures using JANA2006 software [8]. We note that the single-crystal data were obtained up to ∼94 GPa and the images show that under pressure the structure does not change (see Figs. S2 and S3). However, at elevated pressures, particularly above 50 GPa, the quality of the SC data deteriorates. This could be due to two main reasons: 1) The crystal experiences more stress as the pressure medium becomes less hydrostatic. 2) Electronic or structural transitions often induce such lowering of the crystal quality [9], with

the quality possibly improving upon completion of the transition, as seen in this case at 93.9(14) GPa [Figs. S2 and S3]. Correspondingly the error estimates of the atomic positions and unit-cell parameters obtained above 50 GPa are large; accordingly we do not plot them. The powder diffraction data were first integrated using DIOPTAS [10] and then analyzed by LeBail extraction using GSAS II [11]. Powder XRD was used mainly for obtaining the lattice parameters and equations of state (EOS) at the whole pressure range. The pressure uncertainties are ~0.3-1.5 GPa from low to high pressures. Due to the large number of overlapping peaks, the c axis values were fixed according to the position of the (002) peak, which is well separated from other peaks. The reported uncertainties are given according to the standard errors obtained from the respective software used for fitting the data.

Structure solution and refinement of Fe_4O_5

The crystal structure of Fe_4O_5 reported by Lavina *et al.* [12] (space group $Cmcm$, $Z = 4$) was used as a starting model for the refinement. Since a body of the diamond anvil cell shadows more than 50% of the diffraction reflections, the reflection datasets were incomplete. In order to improve the data/parameter ratio, we refined atomic thermal parameters in isotropic approximation. The structures were refined by full-matrix least squares against F^2 using the SHELXL-2014/7 [13] implemented in OLEX2 software [14]. The resulting R_1 values varied from 6.0 to 7.8% The most intense residual electron density peaks were not exceeding 1.8 e/Å^3 and were found within 1 Å of the Fe-atoms. The detailed summary of the crystal structure refinements along with information on unit cell parameters, atomic coordinates and isotropic displacement parameters are summarized in Tables S3–S5.

To evaluate the oxidation states of different Fe ions in the crystal structure we applied a bond-valence-sum (BVS) approach [15] based on the analysis of Fe–O bond lengths around the Fe ions. In this method the oxidation state of an ion V_i is the sum of all its bond valences S_{ij}, each of which is determined as $S_{ij} = exp((R_{ij} – d_{ij})/b_0)$, where d_{ij} is the distance between atoms i and j, R_{ij} is the empirically determined distance for this cation–anion pair and b_0 is an empirical parameter, which is normally about 0.37 Å [15].

Electrical transport measurements

Electrical transport measurements up to 100 GPa were performed using TAU piston-cylinder DACs. A crystal with dimensions of about 70 × 70 × 20 μm was loaded into a cavity of 100 μm in diameter, drilled in a rhenium gasket. The gasket was insulated with a layer of Al_2O_3-NaCl (in the atomic ratio of 3:1) that was mixed with an epoxy. Some amount of a $CaSO_4$ powder was prepressed into the cavity before the crystal loading to ensure a quasi-hydrostatic environment around the sample. Six platinum triangles, which served as electrodes, were placed on the culet. This assembly permitted the measurements in various DC four-probe arrangements at a given pressure. The Pt electrodes were connected to exterior conducting wires with a silver epoxy. The electrical resistance was measured as a function of pressure and temperature (for both compression and decompression cycles) using the standard four-probe method in a custom-made cryostat. At each temperature point, the voltage was measured as a function of a series of applied electrical currents to determine the electrical resistance from a slope of their dependence. The temperature was measured using a Lakeshore Si (DT-421-HR) diode in proximity to the DAC. A few ruby pieces were placed in the central region of the culet between the Pt electrodes around the sample for determination of the pressure. The pressure inside the DAC was determined both before and after each measurement using the ruby R_1 fluorescence line as a pressure marker together with the calibration scales reported in Ref. [16]. The pressure gradients in the DAC were found to be rather insignificant. They amounted to ~5% for the distances across which the voltage was measured (15-20 μm between the tips of the Pt electrodes).

X-ray diffraction results

Our XRD experiments find the conservation of the original orthorhombic $CaFe_3O_5$-type structure up to ~100 GPa (see Figs. S2-S5) but reveal a number of features in its compression behavior. Similar to [17], we find a steeper decrease of the unit-cell volume with pressure increase above ~50 GPa, with $\Delta V/V \sim 2.4\%$. Above 56 GPa the volume variation becomes more gradual and shows only an additional tiny volume drop of ~0.3% at about 84 GPa. Analyzing the $V(P)$ behavior we can highlight the existence of three pressure ranges, namely, from ambient pressure to about 50 GPa (labelled as LP phase), 56–81 GPa (HP1 phase),

and 84–100 GPa (HP2 phase) (see Table S1). As we show in the main text these pressure ranges correspond to different electronic states within the same crystal structure. The unit-cell volume data for these ranges can be fitted using the third- or second-order Birch-Murnaghan (BM3/BM2) equations of state (EOS) [18] (see Table S2). The fit of the $V(P)$ data was performed with the software EosFit GUI [19], using weights for both P and V (see Table S2).

We note a significant "softening" of the material beyond ~50 GPa. Thus, the bulk modulus of Fe_4O_5 almost halved and V_0 increase by ~8%, compared to the LP phase (see Table S2). Such a behavior contradicts with the anticipated hardening of the material after the volume collapse. Similar behavior was reported for $PrFeO_3$ [20], in which a transition in Fe ions to the LS state was not completed simultaneously with a sharp isostructural phase transition around 50 GPa and continued as a sluggish second-order HS-LS transition at higher pressures.

Pressure-induced high axial anisotropy of Fe_4O_5

We note an unusual compression behavior of the parameter b leading to a high axial compression anisotropy of the LP phase (see Fig. S6). In the previous work [2], it was documented that for a single crystal of Fe_4O_5 the parameter b has a negative thermal expansion. This anomalous behavior was tentatively addressed to an antiferromagnetic spin alignment along this axis, which can gain in the exchange energy upon a Fe-Fe distances increase [17, 21]. Such spin alignment may explain also the observed unusual axial anisotropy of the LP phase and indications of the reduced compressibility of the FeO6 octahedra comprising of the Fe2 sites. We note that the high axial anisotropy collapses concurrently with the partial spin transition at ~55 GPa, tentatively as the result of a significant decrease of T_N and H_{hf} accompanying usually HS-LS transitions [22–27].

Temperature dependencies of the electrical resistance of Fe_4O_5

Up to about 86 GPa the temperature dependencies of the electrical resistance of Fe_4O_5 are semiconducting-like, exhibiting a negative $R(T)$ slope (Fig. 2a). The curve at different fixed pressures for certain temperature ranges can be well described by an expression as

follows $lnR = lnR_0 + E_a/k_BT$, where k_B is the Boltzmann's constant, E_a the electrical transport activation energy [see Fig. 3(a)].

We note that a high-pressure low-temperature phase diagram of Fe_4O_5 determined in a previous study [4] established the existence of novel charge-ordered phases at moderate high pressures up to 40 GPa. Hence, the temperature dependencies of the electrical resistance in this pressure range should correspond to a mixture of the room temperature and low-temperature partially charge-ordered phases. The $lnR(1/T)$ dependences for pressures 4.2, 10.7 and 16.5 GPa show a small kink around 240 K, which, according to the phase diagram is consistent with the transition from orthorhombic $Cmcm$ to charge-ordered Fe_4O_5-III phase [4]. In addition, for 10.7 and 16.5 GPa some change of the $lnR(1/T)$ slope is observed around 130 K coinciding with the onset of Fe_4O_5-IV phase [4]. For 4.2 GPa the change is around 110 K coinciding with the transition to Fe_4O_5-II [4]. For $P = 37.2$ and 50 GPa there is only a slope change around 230 K and at 61.2 GPa no appreciable changes are observed in $lnR(1/T)$ behavior.

Theoretical Methods

We supplement our single-crystal (SC) and powder (PWD) synchrotron x-ray diffraction (XRD) and resistance measurements by the density functional theory+dynamical mean-field theory (DFT+DMFT) electronic structure calculations [28, 29]. We study the electronic structure, magnetic properties, and valence configurations of iron of paramagnetic Fe_4O_5 using the fully charge self-consistent DFT+DMFT method implemented within plane-wave pseudopotentials [25, 26]. By using DMFT it becomes possible to treat the effects of electronic correlations (e.g., local spin and charge fluctuations) on the electronic properties of strongly correlated electron materials near the Mott transition [22, 23, 25, 26, 28–30]. In our DFT+DMFT calculations we construct a basis set of atomic-centered symmetry-constrained Wannier functions for the partially occupied Fe $3d$ bands evaluated within DFT [31]. The DFT+DMFT calculations are performed in the local basis set determined by diagonalization of the corresponding Fe $3d$ occupation matrices. We used the continuous-time hybridization-expansion quantum Monte-Carlo (segment) algorithm to solve the realistic many-body problem [32]. The DFT+DMFT calculations are performed in the *paramagnetic* state at an electronic temperature of $T_{el} \sim 770$ K (our calculations at 580 K give quanti-

tively similar results). In DMFT we use the Hubbard interaction $U = 6$ eV and Hund's exchange coupling $J = 0.89$ eV for the Fe $3d$ states in accordance with previous estimates [22, 23, 25, 26]. The U and J values are assumed to remain constant upon variation of the lattice. The Coulomb interaction was treated in the density-density approximation, spin-orbit coupling was neglected.

The electronic structure and magnetic properties of Mott-Hubbard systems are known to depend sensitively on the choice of the Coulomb interaction [33, 34]. We therefore check different Hubbard U values, to make our DFT+DMFT based conclusions robust. In particular, we performed DFT+DMFT calculations with a sufficiently smaller Hubbard parameter $U = 5$ eV (while using the same Hund's coupling, $J = 0.89$ eV). The DFT+DMFT approach was implemented within the plane-wave pseudopotentials with the generalized gradient approximation in DFT. We employed the fully localized double-counting correction, evaluated from the self-consistently determined local occupancies, to account for the electronic interactions already described by DFT. The spectral functions were computed using the maximum entropy method. Further technical details about the method used can be found in Ref. [25, 26].

In our DFT+DMFT calculations we adopted the crystal structure parameters taken from SC-XRD at three distinct pressures, corresponding to three distinct electronic phases in the equation of states of room-temperature Fe_4O_5, at ∼39 GPa, 62 GPa, and 84 GPa. While at 62 and 84 GPa the quality of SC-XRD data were not sufficient to extract atomic positions (only the lattice parameters were determined), we used atomic positions (internal atomic coordinates) obtained at 46.8 GPa for the DFT+DMFT calculations at 62 and 84 GPa. We use the DFT+DMFT approach with three nonequivalent impurity sites in the unit-cell in order to treat electron correlations in the Fe $3d$ bands of the crystallographically distinct Fe1 (with prismatic oxygen coordination) and Fe2 and Fe3 ions (with octahedral coordination) of the orthorhombic $CaFe_3O_5$-type structure with space group $Cmcm$. For the given phases, we computed the site and orbitally-resolved Fe $3d$ spectral functions, performed analysis of oxidation state of Fe ions, evaluated local spin susceptibilities $\chi(\tau) = \langle \hat{m}_z(\tau)\hat{m}_z(0)\rangle$, instantaneous and fluctuating local magnetic moments, $M_{\rm loc}$. The latter are evaluated as the imaginary-time average of local spin susceptibilities as $M_{\rm loc} = \left(k_{\rm B}T\int\chi(\tau)d\tau\right)^{1/2}$. Our results for the pressure-dependence of the site-resolved $\chi(\tau)$ of Fe_4O_5 are shown in Fig. S7.

In agreement with our resistance measurements our DFT+DMFT calculations predict an

activation type behavior of the temperature-dependent resistance below ∼84 GPa. Above ∼84 GPa it undergoes a site-selective Mott IMT associated with metallization of the octahedral Fe2 and Fe3 sites, while the prismatic Fe1 sites remain Mott localized; thus, above ∼84 GPa our calculations predict metallization of Fe_4O_5, in agreement with experiment. Moreover, our results for an energy gap are compatible to that extracted from our resistance measurement (taking into account that the energy gap depends on the Hubbard U value). In addition, both experiment and theory show a gradual decrease of an energy gap value under pressure (which is typical for the high-pressure behavior of the Mott-Hubbard systems).

To determine the valence state of iron, we computed the reduced density matrix of the Wannier Fe 3d states within DMFT. The eigenvalues of the reduced Fe 3d density matrix give a probability of observing different 3d electron atomic configurations, i.e., the Fe 3d electrons are seen fluctuating due to quantum and temperature effects between various atomic configurations (see Ref. [25, 26] for further details). We note that this quantum many-body Wannier basis set based definition of valence gives an upper estimate. Consistent with this, our analysis yields nearly "pure" (Wannier) Fe 2+ and 3+ configurations for the prismatic and octhahedral iron sites in Fe_4O_5 at low pressures. At the same time, the valence configuration can be approximated as an integrated charge density inside atomic sphere with a given radius (in accordance with quantum chemical Bader's charge analysis). This quantum-chemical estimate of the oxidation state is related to bond valence sum analysis [15], in which valence is characterized by the bond length and coordination of an atom (here, prismatic or octahedral oxygen coordination). It is known to give a lower bound estimate of the valence state and hence of charge disproportionation (see, e.g., Refs. [35–37]). We note that both estimates of the oxidation state of iron in Fe_4O_5 yield qualitatively consistent results, giving direct evidence for charge ordering in room-temperature Fe_4O_5. Moreover, the quantum-chemical estimate of charge disproportionation of Fe ions in Fe_4O_5 gives about 0.14 $\bar{e}$ charge difference (i.e., of ∼14% of the ideal valence skipping). This value is consistent with previous estimates for the low-temperature charge-ordered phases of the mixed-valent oxides, such as Fe_3O_4, of ∼0.2 [35–37], and high-pressure charge-disproportionated phase of Fe_2O_3, of ∼0.1-0.18 [22, 23].

Overall, our results for the electronic and lattice properties of the $Cmcm$ phase of Fe_4O_5 agree well with experimental data and provide a microscopic understanding of the complex pressure-induced evolution of the electronic and magnetic properties of Fe_4O_5. In particu-

lar, we obtain that at low pressures and room temperature Fe_4O_5 is a narrow-gap correlated insulator with an orthorhombic $CaFe_3O_5$-type crystal structure characterized by a localized charge ordering behavior of the Fe 2+/3+ cations with alternating arrangement of iron (010) planes occupied by Fe 2+/3+ cations. This result is consistent with an activation type behavior of the temperature-dependent resistance for pressures below ∼86 GPa (below 298 K), implying the formation of the Verwey-type charge ordering in Fe_4O_5. Under pressure, Fe_4O_5 undergoes a series of complicated isostructural electronic and magnetic-state transformations (space group symmetry remains unchanged, *Cmcm*). First, a HS-LS transition sets in for a fraction of the octahedral Fe^{2+} ions (only the Fe2 sites are involved) starting at ∼50 GPa, which is followed by the site-selective Mott insulator-to-metal phase transition with metallization of the octahedral Fe2 and Fe3 states and Fe 2+ to 3+ valence crossover at the prismatic Fe sites (above 84 GPa). Thus, above ∼86 GPa the octahedral Fe2 and Fe3 sites adopt the LS state and are metallic, while the prismatic Fe1 sites are in the HS state and are Mott insulating.

While the DFT+DMFT calculations of the electronic structure and structural phase stability of Fe_4O_5 are computationally very demanding, we perform more simplified spin-polarized DFT electronic structure calculations of the compressional behavior of Fe_4O_5. In DFT we use the generalised gradient approximation for the correlation exchange functional [38]. To this end, we adopt the crystal structure data of RT Fe_4O_5 taken by our single crystal XRD at 39 GPa and calculate the total energy of antiferromagnetically ordered state of Fe_4O_5 as a function of volume. While such calculations cannot explain the Mott insulating state and, hence, underestimate orbital ordering and charge ordering/disproportionation effects in Fe_4O_5, DFT predicts two consecutive pressure-induced HS-LS transitions at the octahedral Fe sites, first at Fe2 at ∼58 GPa and then at Fe3 above 80 GPa (see Figs. S8 and S9). At the same time, the prismatic Fe1 ions are in the HS state up to high pressure of ∼150 GPa. This result agrees well with the compressional behavior of Fe_4O_5 obtained by using the more sophisticated DFT+DMFT method. We note that DFT calculations predict a sizable *decrease* of the bulk modulus at ∼58 GPa from K_0 = 172 GPa for the LP phase to ∼140 GPa for the HP1 phase which is associated with the HS-LS crossover at the octahedral Fe2 sites, in agreement with our experimental results. The phase transition is accompanied by a volume collapse of ∼3.5%. Upon further compression, above ∼80 GPa our DFT calculations predict a HS-LS crossover at the octahedral Fe3 sites. It is accompanied by a reduction of

the unit-cell volume by $\sim$1.5% and by a sizable *increase* of the bulk modulus from 140 GPa (in the HP1 phase) to $\sim$176 GPa (in the HP2 phase). We note however that DFT calculations cannot properly explain a Fe^{2+} to Fe^{3+} valence crossover at the prismatic Fe1 sites at high pressure which is according to our DFT+DMFT results is related to the site-selective Mott IMT (metallization of the octahedral Fe2 and Fe3 sites, while the prismatic Fe1 sites remain Mott localized). This implies the crucial importance of the effects of strong electronic correlation, consistent with previous studies of the site-selective Mott IMT in Fe_2O_3 and $R\mathrm{NiO}_3$ compounds.

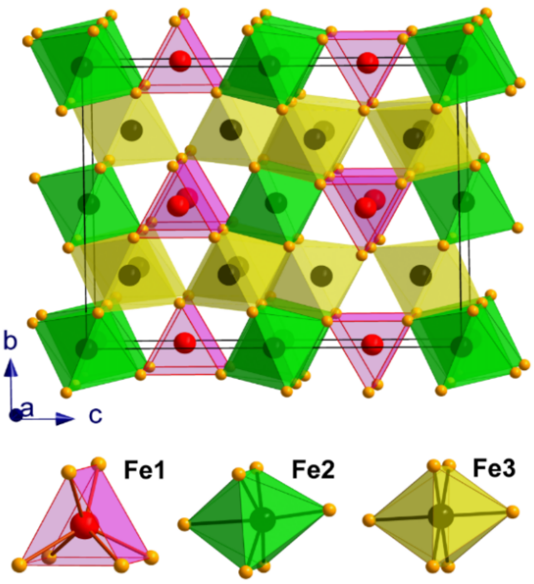

FIG. S1: Crystal structure of Fe_4O_5 at ambient conditions.

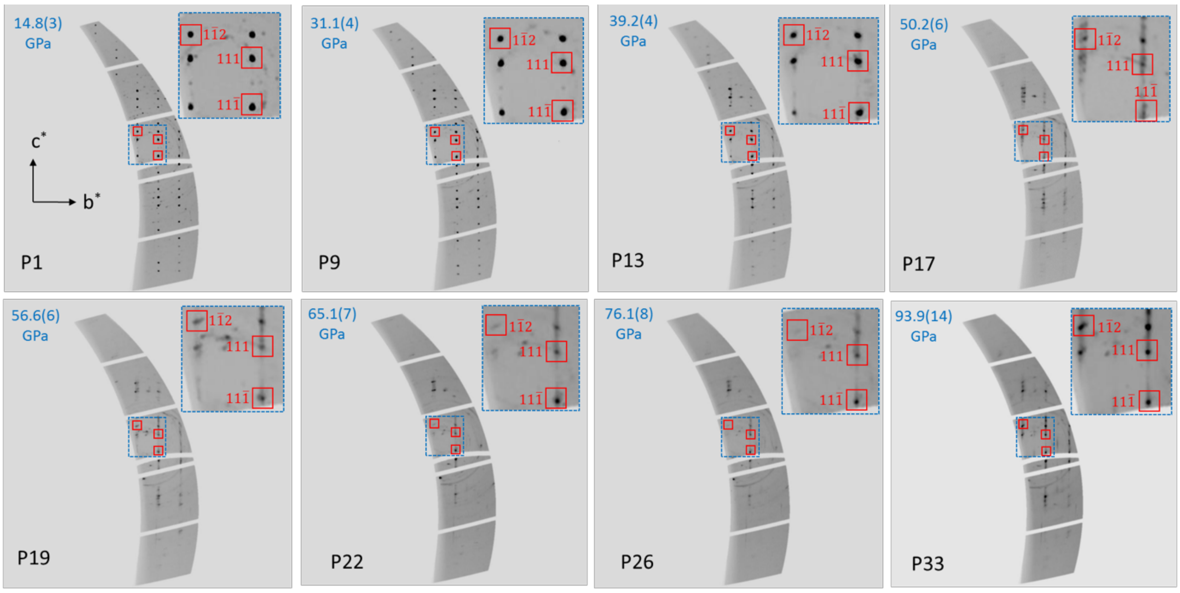

FIG. S2: Single-crystal X-ray diffraction patterns of Fe_4O_5 at RT at various pressures. Reconstructed reciprocal lattice plane defined by [001] and [010] directions of a selected Fe_4O_5 grain at various pressures shown in the $1kl$ plane. Insets show an enlarged portion.

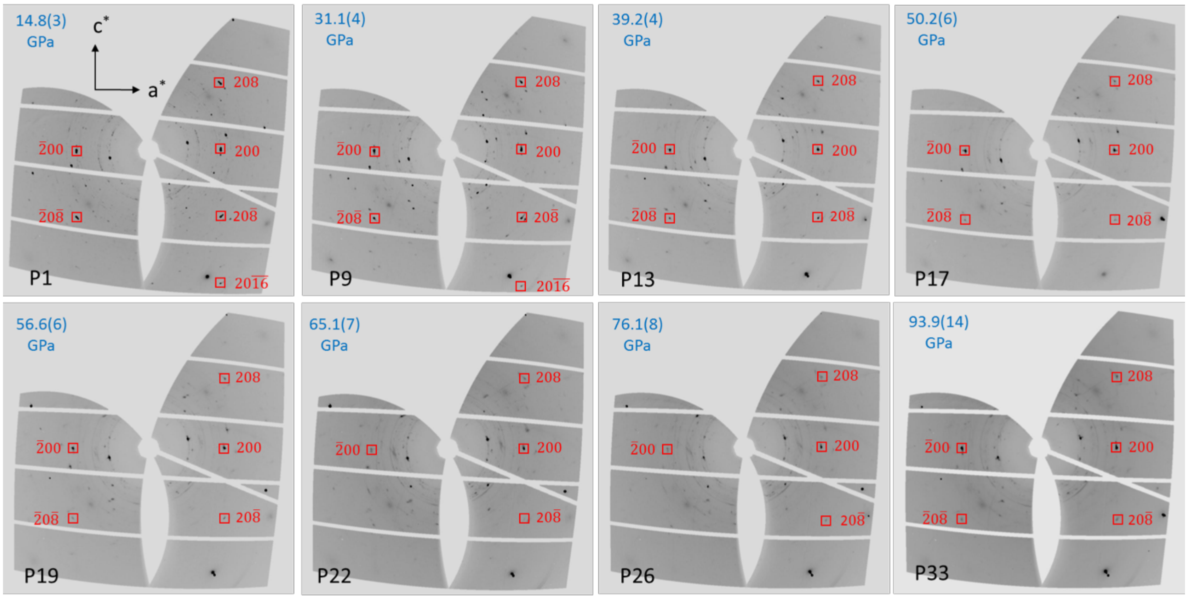

FIG. S3: Reconstructed reciprocal lattice plane defined by [001] and [100] directions of a selected Fe_4O_5 grain at various pressures shown in the $h0l$ plane.

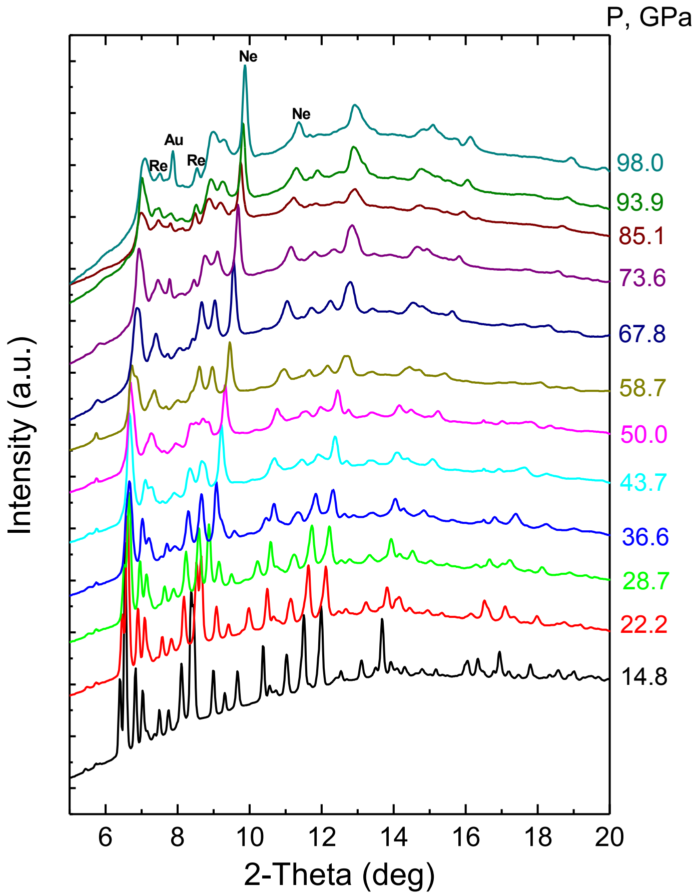

FIG. S4: X-ray powder diffraction patterns of Fe_4O_5 at RT at various pressures ($\lambda = 0.2952$ Å).

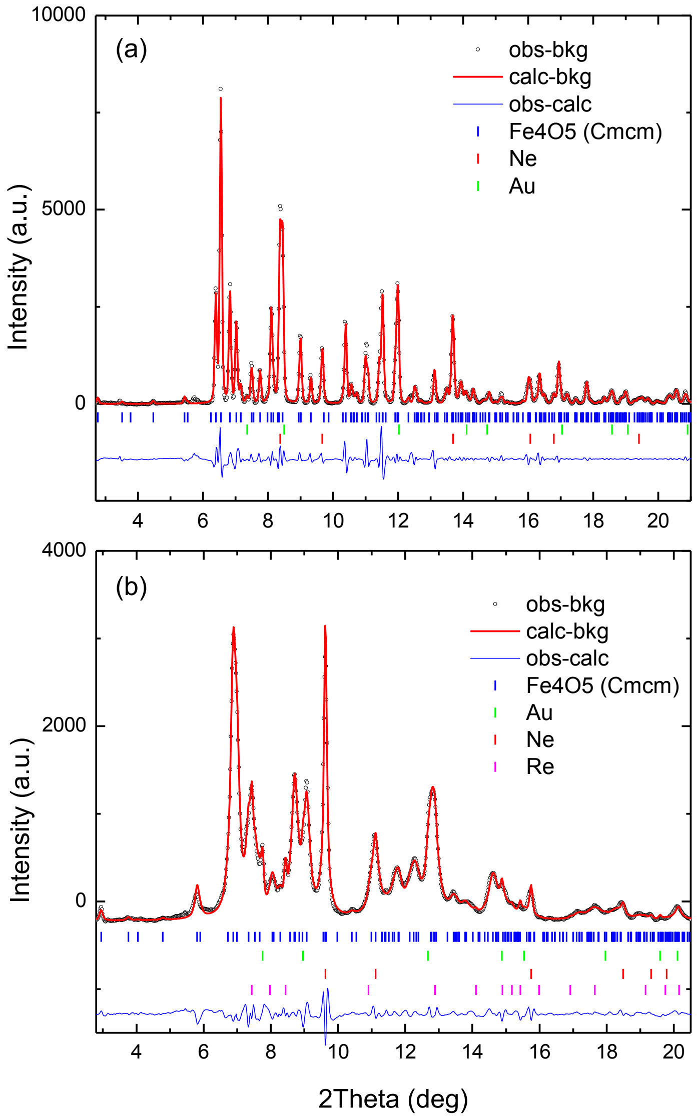

FIG. S5: Refinement of XRD patterns collected at $T = 300$ K for Fe_4O_5 at 14.8 (a) and 73.6 GPa (b), respectively and the differences between the observed and calculated profiles. Marks show the calculated peak positions.

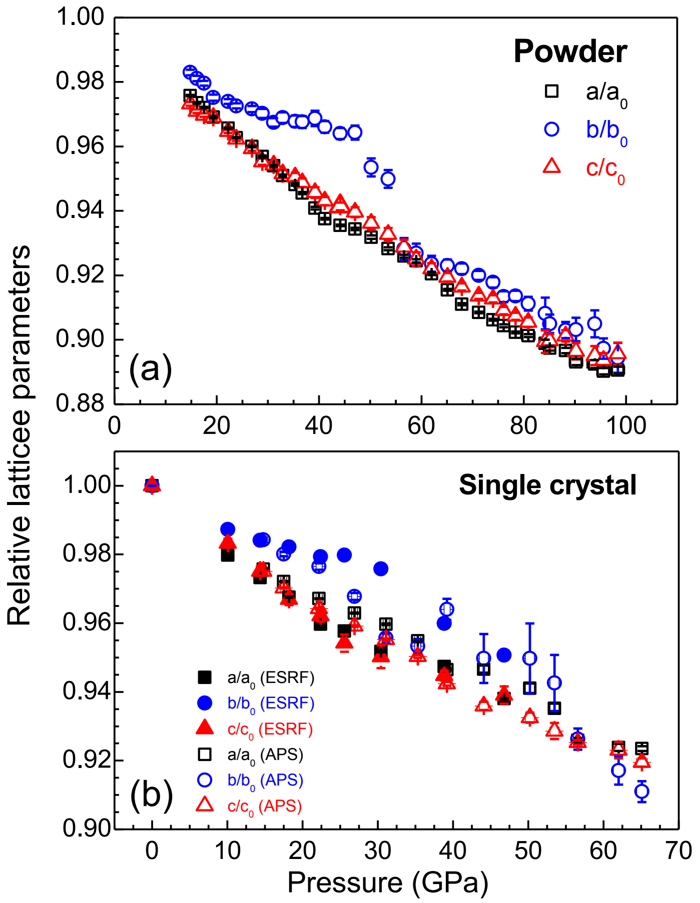

FIG. S6: Pressure dependencies of the relative unit-cell parameters, determined in the powder (a) and single crystal (b) X-ray diffraction studies. Note that parameters a and c show similar behavior and almost do not exhibit any peculiarities over the entire pressure range up to 100 GPa, except a small drop of the parameter c at ∼84 GPa (see Fig. 1a). Meanwhile, the behavior of the parameter b varies with pressure more dramatically: it shows a high stiffness and in the range of about 25–40 GPa it almost does not shrink under pressure. At higher pressures, the b parameter shows a steeper decrease with pressure increase followed by a drop in the range of 53–56 GPa and only above 56 GPa it behaves similar to two other lattice parameters indicating the collapse of the axial anisotropy.

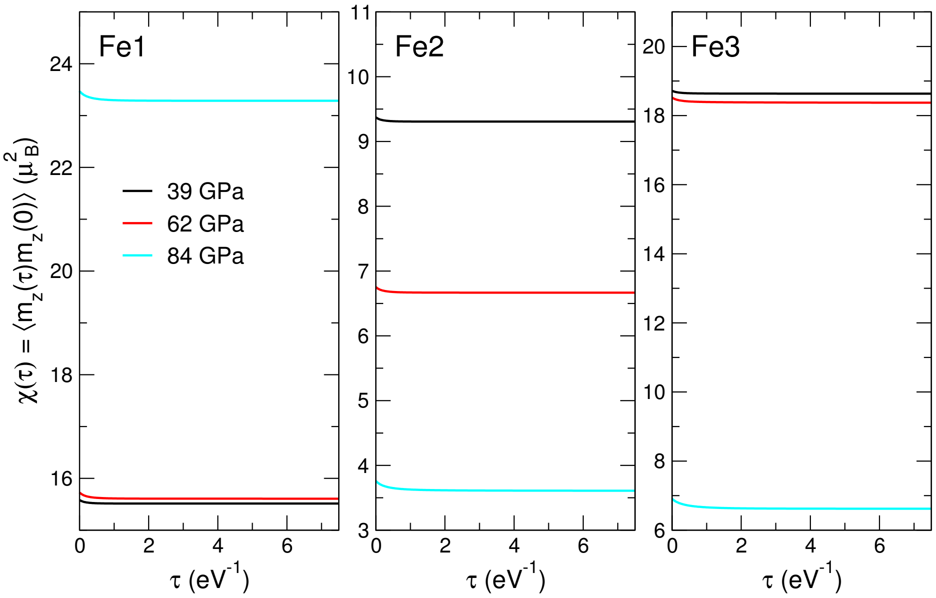

FIG. S7: Site-resolved local spin susceptibilities $\chi(\tau) = \langle \hat{m}_z(\tau)\hat{m}_z(0)\rangle$ of paramagnetic Fe_4O_5 as obtained by DFT+DMFT for different pressures.

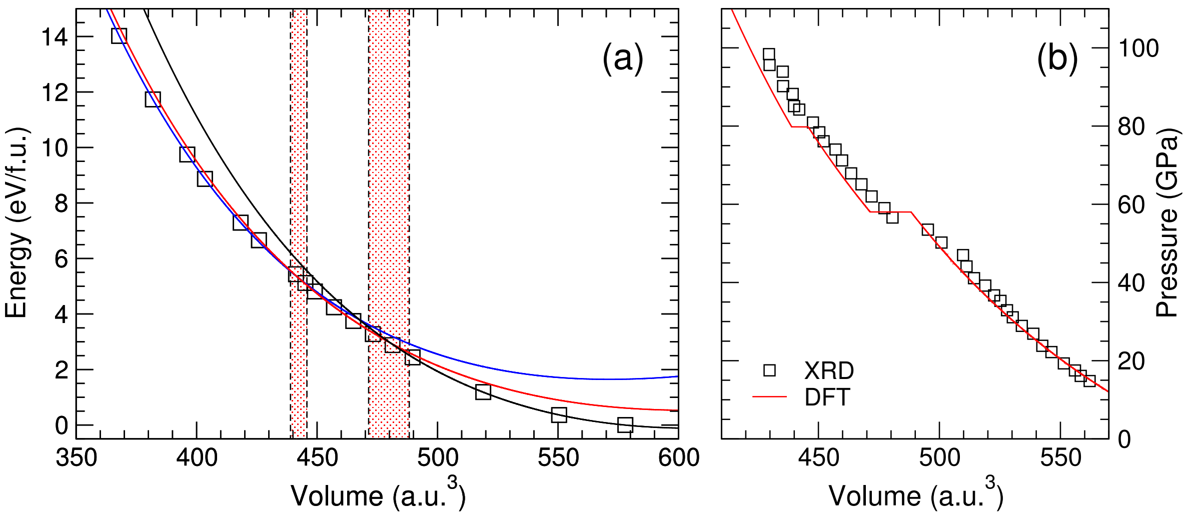

FIG. S8: Compressional behavior of Fe_4O_5 obtained by spin-polarized DFT total-energy calculations: the DFT total-energy results (a) and the obtained equation of states in comparison to XRD data (b). In (a) the DFT total-energy results are depicted by black squares. Our DFT results for two consecutive HS-LS transitions at the octahedral Fe2 (at $V \sim 488.3$ a.u.^{3}, at $\sim$58 GPa) and Fe3 ions (at $V \sim 445.8$ a.u.^{3}, at $\sim$80 GPa) and the corresponding collapse of the lattice volume of 3.5% and 1.5%, respectively, are shown with red shadings. Different colors depict different sets of EOS data. In (b) the XRD V-P data points are shown by black squares.

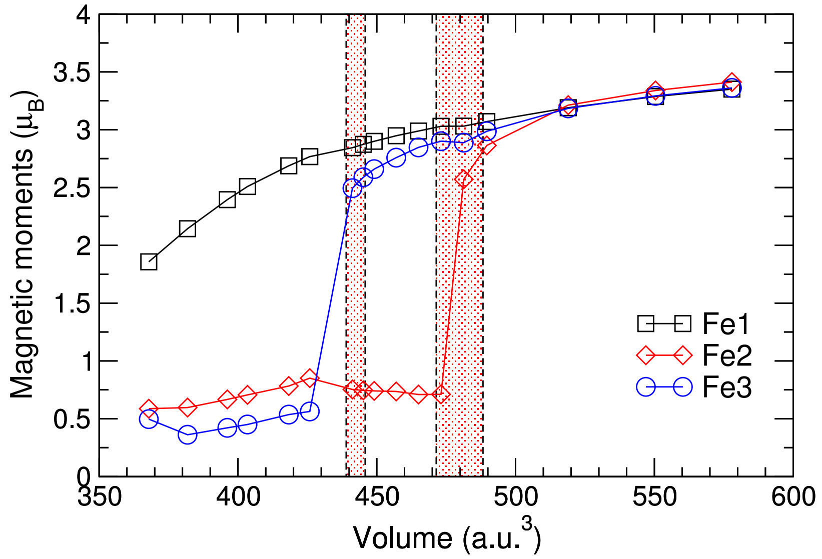

FIG. S9: Compressional behavior of magnetic moments of the prismatic Fe1 and octahedral Fe2 and Fe3 ions as obtained by spin-polarized DFT calculations. Our DFT results for two consecutive HS-LS transitions at the octahedral Fe2 (at $V \sim 488.3$ a.u.^{3}, at ∼58 GPa) and Fe3 ions (at $V \sim 445.8$ a.u.^{3}, at ∼80 GPa) and the corresponding collapse of the lattice volume of 3.5% and 1.5%, respectively, are shown by red rectangles.

Table S1: The refined unit-cell volume of the *Cmcm* phase of Fe_4O_5 at various pressures. The uncertainties in the unit-cell volume are solely from the GSAS fitting output. Notes: the data points at the LP-HP1 transition range between 50 and 56 GPa were not used in any of the EOSs. For the LP EOS region we also used the ESRF single crystal data (except the point at 46.8 GPa). The R_{wp} is the weighted-profile R-factor: $R_{wp} = \sqrt{\frac{\sum w_i(y_i(obs)-y_i(calc))^2}{\sum w_i y_i(obs)^2}}$.

EOS	P (GPa)	V (Å^3)	R_{wp} (%)
LP	14.8(3)	333.15(9)	0.90
LP	16.1(3)	330.97(9)	0.85
LP	17.5(4)	329.56(8)	0.75
LP	19.3(4)	326.83(9)	0.78
LP	22.2(4)	323.79(7)	0.62
LP	23.8(4)	321.56(8)	0.59
LP	26.9(4)	319.36(10)	0.66
LP	28.9(4)	316.54(14)	0.90
LP	31.1(4)	314.33(13)	0.84
LP	32.9(6)	312.92(17)	0.83
LP	35.3(4)	311.31(18)	0.86
LP	36.7(4)	309.76(18)	0.80
LP	39.2(4)	307.60(22)	0.84
LP	41.1(5)	304.84(17)	0.63
LP	44.1(6)	303.00(16)	0.58
LP	47.0(6)	302.15(20)	0.71
	50.2(6)	296.87(26)	0.86
	53.5(6)	293.54(25)	0.73
HP1	56.6(6)	284.88(29)	0.73
HP1	59.0(7)	282.90(28)	0.76
HP1	62.0(7)	279.77(21)	0.60
HP1	65.1(7)	277.28(16)	0.47
HP1	67.9(7)	274.75(14)	0.42
HP1	71.2(7)	272.53(12)	0.35
HP1	74.0(8)	270.93(14)	0.38
HP1	76.1(8)	268.01(19)	0.53
HP1	78.4(8)	266.94(15)	0.37
HP1	80.9(12)	265.39(21)	0.45
HP2	84.2(10)	262.04(50)	0.63
HP2	85.1(10)	260.77(30)	0.47
HP2	88.2(10)	260.43(32)	0.45
HP2	90.2(12)	258.08(40)	0.59
HP2	93.9(14)	258.01(49)	0.85
HP2	95.6(12)	254.77(37)	0.61
HP2	98.4(12)	254.62(45)	0.61

Table S2: Birch-Murnaghan EOS for the various phases of Fe_4O_5. (K_0, K' and V_0 are the bulk modulus, its pressure derivative, and the unit-cell volume (at 1 bar and 300 K), respectively; K_{50} and K_{84} are the bulk modulus at 50 and 84 GPa, correspondingly). We note that the values obtained for LP phase are in good agreement with previous data [7,9,10].

Phase	EOS	V_0, Å^3	K_0, GPa	K_{50}, GPa	K_{84}, GPa	K'
LP phase, 0 – 50 GPa	BM3	356.88(3)	175(3)	417(3)		5.4(3)
LP phase, 0 – 50 GPa	BM2	356.86(5)	190.2(11)	375(1)		4
HP1 phase, 56 - 81 GPa	BM2	386(7)	102(9)	279(3)	388(11)	4
HP1 phase, 56 –81 GPa	BM3	370(60)	150(200)	293(8)	371(8)	3(3)
HP2 phase, 84 – 100 GPa	BM2	360(26)	140(40)		430(60)	4

Table S3. Details of crystal structure refinements for Fe_4O_5 at high pressures* (ESRF)

Pressure, GPa	0.0001	10.1(5)	14.4(5)	18.2(5)	22.4(5)	25.6(5)	30.4(5)	31.7(5)	46.8(5)
a (Å)	2.89510(13)	2.83673(19)	2.8176(2)	2.8012(4)	2.7785(4)	2.7726(6)	2.7554(7)	2.7601(6)	2.7158(5)
b (Å)	9.8054(6)	9.6803(8)	9.6494(10)	9.6307(15)	9.6029(15)	9.607(2)	9.568(3)	9.589(3)	9.3216(18)
c (Å)	12.576(9)	12.366(15)	12.263(16)	12.16(2)	12.10(2)	12.00(3)	11.95(4)	11.84(4)	11.81(3)
V (Å^3)	357.0(3)	339.6(4)	333.4(4)	327.9(6)	322.9(6)	319.6(9)	315.1(10)	313.5(10)	298.9(7)
Reflections collected	416	382	403	355	384	294	283	348	305
Independent reflections	79	80	77	77	76	76	71	77	67
Independent reflections [$I > 2\sigma(I)$]	75	74	73	75	71	70	64	71	58
Refined parameters	15	15	15	15	15	15	15	15	15
$R_{int}(F^2)$	0.0409	0.0439	0.0481	0.0422	0.04	0.0364	0.0684	0.0597	0.0699
$R(\sigma)$	0.0215	0.0375	0.0219	0.0175	0.0193	0.0179	0.0485	0.0337	0.0501
R_1 [$I > 2\sigma(I)$]	0.0651	0.06	0.0563	0.0711	0.0775	0.0717	0.0625	0.0755	0.0743
wR_2 [$I > 2\sigma(I)$]	0.1702	0.15	0.1603	0.1898	0.189	0.163	0.1682	0.1884	0.1921
R_1	0.0658	0.0617	0.0571	0.0719	0.0794	0.0742	0.0666	0.0783	0.0777
wR_2	0.1743	0.1571	0.1664	0.1913	0.1984	0.1701	0.1723	0.1912	0.1977
Goodness of fit on F^2	1.161	1.218	1.212	1.211	1.139	1.115	1.208	1.138	1.126
$\Delta\rho_{max}(e$ / Å$^3)$	1.487	1.235	0.732	1.187	1.797	1.268	0.866	1.199	1.169
$\Delta\rho_{min}(e$ / Å$^3)$	-1.416	-1.243	-1.418	-1.474	-1.556	-1.083	-0.89	-1.434	-1.142

Table S4. Structural data for Fe_4O_5 at high pressures* (ESRF)

Pressure, GPa	0.0001	10.1(5)	14.4(5)	18.2(5)	22.4(5)	25.6(5)	30.4(5)	31.7(5)	46.8(5)
y (Fe1)	0.5054(3)	0.5038(3)	0.5039(3)	0.5047(4)	0.5051(3)	0.5058(3)	0.5069(4)	0.5075(4)	0.5092(4)
y (Fe3)	0.26023(17)	0.26144(18)	0.2620(2)	0.2627(2)	0.2631(2)	0.26352(19)	0.2643(3)	0.2647(3)	0.2659(3)
z (Fe3)	0.1182(3)	0.1179(4)	0.1182(4)	0.1178(6)	0.1185(5)	0.1181(5)	0.1177(8)	0.1182(7)	0.1185(7)
y (O1)	0.1593(13)	0.1588(15)	0.1582(16)	0.1598(19)	0.1606(19)	0.1592(15)	0.1577(16)	0.1618(16)	0.161(2)
y (O2)	0.3580(8)	0.3579(9)	0.3569(9)	0.3582(12)	0.3569(12)	0.3593(9)	0.3578(13)	0.3553(13)	0.3639(12)
z (O2)	0.543(2)	0.546(2)	0.543(3)	0.545(3)	0.547(3)	0.549(3)	0.543(3)	0.546(3)	0.548(4)
y (O3)	0.0929(9)	0.0899(10)	0.0891(11)	0.0887(13)	0.0857(14)	0.0883(12)	0.0866(14)	0.0856(14)	0.0851(17)
z (O3)	0.6416(16)	0.6429(19)	0.6430(18)	0.643(3)	0.643(2)	0.647(2)	0.649(3)	0.647(2)	0.650(3)
U_{iso}(Fe1) (Å^2)	0.0084(10)	0.0071(9)	0.0088(10)	0.0080(11)	0.0132(12)	0.0110(10)	0.0112(12)	0.0097(12)	0.0216(14)
U_{iso}(Fe2) (Å^2)	0.0088(10)	0.0086(9)	0.0103(10)	0.0099(12)	0.0152(12)	0.0118(11)	0.0131(12)	0.0121(12)	0.0262(14)
U_{iso}(Fe3) (Å^2)	0.0077(9)	0.0074(8)	0.0092(9)	0.0086(11)	0.0140(11)	0.0115(10)	0.0116(11)	0.0110(11)	0.0244(13)
U_{iso}(O1) (Å^2)	0.010(2)	0.010(2)	0.012(3)	0.009(3)	0.015(3)	0.008(2)	0.007(3)	0.005(3)	0.018(3)
U_{iso}(O2) (Å^2)	0.011(2)	0.010(2)	0.013(2)	0.011(3)	0.018(3)	0.011(2)	0.010(2)	0.009(2)	0.020(3)
U_{iso}(O3) (Å^2)	0.0104(18)	0.0082(19)	0.0091(19)	0.007(2)	0.015(2)	0.010(2)	0.009(2)	0.007(2)	0.020(2)

*atoms' Wyckoff positions:
Fe1 4*c* (0, *y*, 0.25)
Fe2 4*a* (0, 0, 0)
Fe3 8*f* (0, *y*, *z*)
O1 4*c* (0, *y*, 0.25)
O2 8*f* (0, *y*, *z*)
O3 8*f* (0, *y*, *z*)

Table S5. Interatomic distances in FeO6 polyhedra in Fe_4O_5 (ESRF)

**Pressure, GPa**	**0.0001**	**10.1(5)**	**14.4(5)**	**18.2(5)**	**22.4(5)**	**25.6(5)**	**30.4(5)**	**31.7(5)**	**46.8(5)**
***d*(Fe1−O1) x2 (Å)**	2.091(9)	2.064(11)	2.049(11)	2.047(14)	2.040(14)	2.023(11)	1.995(11)	2.023(11)	1.960(15)
***d*(Fe1−O3) x4 (Å)**	2.210(13)	2.142(14)	2.124(14)	2.11(2)	2.087(19)	2.066(15)	2.035(19)	2.047(18)	2.004(18)
***d*(Fe2−O2) x4 (Å)**	2.079(8)	2.055(9)	2.043(10)	2.032(13)	2.035(13)	2.023(11)	2.004(12)	2.031(12)	1.942(14)
***d*(Fe2−O3) x2 (Å)**	2.000(19)	1.97(2)	1.95(2)	1.93(3)	1.92(3)	1.96(2)	1.97(3)	1.92(3)	1.94(3)
***d*(Fe3−O1) (Å)**	1.930(7)	1.912(9)	1.902(9)	1.888(12)	1.871(11)	1.873(10)	1.882(13)	1.847(12)	1.835(14)
***d*(Fe3−O2) (Å)**	2.24(2)	2.23(3)	2.18(3)	2.18(4)	2.20(4)	2.20(3)	2.12(3)	2.13(3)	2.16(4)
***d*(Fe3−O2) x2 (Å)**	2.084(11)	2.035(13)	2.036(15)	2.024(19)	2.002(19)	2.002(14)	2.014(18)	1.991(18)	2.00(2)
***d*(Fe3−O3) x2 (Å)**	2.063(7)	2.044(8)	2.035(8)	2.025(10)	2.033(11)	2.017(9)	2.019(10)	2.020(10)	1.977(12)

Table S6. Details of crystal structure refinements for Fe_4O_5 at high pressures* (APS)

Pressure, GPa	14.8(3)	17.5(4)	22.2(4)	26.9(4)	31.1(4)	35.3(4)	35.3(4)	39.2(4)	44.1(6)	56.6(6)
a (Å)	2.8282(4)	2.8138(5)	2.7994(4)	2.7872(5)	2.7779(7)	2.7632(11)	2.7642(11)	2.7396(12)	2.740(3)	2.680(3)
b (Å)	9.639(5)	9.608(6)	9.572(9)	9.487(10)	9.368(13)	9.35(2)	9.34(2)	9.45(3)	9.31(7)	9.077(14)
c (Å)	12.266(2)	12.204(2)	12.128(3)	12.064(3)	12.015(4)	11.951(5)	11.955(5)	11.853(7)	11.772(15)	11.638(12)
V (Å^3)	334.39(19)	329.9(2)	325.0(3)	319.0(4)	312.7(5)	308.7(7)	308.8(7)	306.8(10)	300(2)	283.1(6)
Reflections collected	480	413	456	404	443	423	429	419	315	263
Independent reflections	179	150	234	216	211	123	127	120	44	60
Independent reflections [$I > 2\sigma(I)$]	232	222	169	153	169	156	160	153	115	90
Refined parameters	15	15	15	15	15	15	15	15	15	15
$R_{int}(F^2)$	0.008	0.0125	0.0207	0.0145	0.0198	0.029	0.0293	0.0383	0.1266	0.0813
$R(\sigma)$	0.0093	0.0134	0.0167	0.0154	0.0155	0.0374	0.0355	0.042	0.094	0.0378
R_1 [$I > 2\sigma(I)$]	0.0394	0.0378	0.0475	0.0478	0.0485	0.0692	0.0655	0.0727	0.1499	0.1055
wR_2 [$I > 2\sigma(I)$]	0.0488	0.0452	0.0536	0.0591	0.0552	0.0765	0.0702	0.0792	0.1607	0.1096
R_1	0.0484	0.0471	0.0522	0.0534	0.0582	0.0832	0.0784	0.1027	0.2395	0.1348
wR_2	0.0497	0.0459	0.0539	0.0596	0.056	0.0781	0.072	0.0815	0.186	0.1112
Goodness of fit on F^2	3	2.49	2.89	3.29	3.12	2.98	2.57	3.05	3.1	2.99
$\Delta\rho_{max}(e$ / Å$^3)$	1.1	1	1.17	1.39	2.32	2.26	1.95	2.4	3.91	1.54
$\Delta\rho_{min}(e$ / Å$^3)$	-1.33	-1.18	-1.73	-1.38	-1.46	-1.63	-1.47	-1.49	-3.91	-1.9

Table S7. Structural data for Fe_4O_5 at high pressures* (APS)

Pressure, GPa	14.8(3)	17.5(4)	22.2(4)	26.9(4)	31.1(4)	35.3(4)	35.3(4)	39.2(4)	44.1(6)	56.6(6)
y (Fe1)	0.5033(2)	0.5037(2)	0.5040(4)	0.5055(4)	0.5060(4)	0.5062(6)	0.5056(6)	0.5063(8)	0.508(4)	0.527(2)
y (Fe3)	0.26186(15)	0.26227(14)	0.2632(2)	0.2637(3)	0.2647(3)	0.2650(4)	0.2645(4)	0.2658(5)	0.265(2)	0.2775(13)
z (Fe3)	0.11790(6)	0.11797(7)	0.11782(10)	0.11790(11)	0.11784(11)	0.11790(17)	0.11781(17)	0.1177(2)	0.1185(7)	0.1093(4)
y (O1)	0.1550(12)	0.1565(10)	0.1555(17)	0.1590(19)	0.1591(18)	0.161(3)	0.163(3)	0.157(4)	0.139(17)	0.207(11)
y (O2)	0.3600(9)	0.3574(7)	0.3582(12)	0.3572(14)	0.3580(13)	0.358(2)	0.357(2)	0.355(2)	0.342(10)	0.353(9)
z (O2)	0.5443(3)	0.5445(4)	0.5442(5)	0.5440(6)	0.5447(5)	0.5446(8)	0.5461(8)	0.5454(10)	0.549(4)	0.547(3)
y (O3)	0.0917(8)	0.0898(7)	0.0888(12)	0.0895(13)	0.0870(13)	0.086(2)	0.0894(19)	0.084(2)	0.091(7)	0.067(5)
z (O3)	0.6445(3)	0.6445(3)	0.6452(5)	0.6456(5)	0.6464(5)	0.6470(7)	0.6474(7)	0.6474(10)	0.647(2)	0.648(2)
U_{iso}(Fe1) (Å^2)	0.0084(3)	0.0073(3)	0.0071(4)	0.0105(5)	0.0097(5)	0.0127(7)	0.0122(7)	0.0134(8)	0.037(4)	0.032(3)
U_{iso}(Fe2) (Å^2)	0.0092(3)	0.0086(3)	0.0082(5)	0.0118(5)	0.0112(5)	0.0140(7)	0.0144(7)	0.0150(9)	0.018(3)	0.025(2)
U_{iso}(Fe3) (Å^2)	0.0088(3)	0.0081(3)	0.0078(4)	0.0114(4)	0.0106(4)	0.0140(6)	0.0137(6)	0.0146(7)	0.019(2)	0.029(2)
U_{iso}(O1) (Å^2)	0.0081(11)	0.0087(12)	0.0061(16)	0.0102(17)	0.0080(17)	0.011(2)	0.011(2)	0.015(3)	0.036(17)	0.038(11)
U_{iso}(O2) (Å^2)	0.0093(8)	0.0080(8)	0.0078(11)	0.0115(12)	0.0101(12)	0.0135(18)	0.0125(19)	0.013(2)	0.028(11)	0.043(8)
U_{iso}(O3) (Å^2)	0.0075(8)	0.0078(8)	0.0071(11)	0.0086(12)	0.0098(12)	0.0103(17)	0.0082(16)	0.012(2)	0.005(6)	0.023(7)

*atoms' Wyckoff positions:
Fe1 4*c* (0, *y*, 0.25)
Fe2 4*a* (0, 0, 0)
Fe3 8*f* (0, *y*, *z*)
O1 4*c* (0, *y*, 0.25)
O2 8*f* (0, *y*, *z*)
O3 8*f* (0, *y*, *z*)

Table S8. Interatomic distances in FeO6 polyhedra in Fe_4O_5 (APS)

**Pressure, GPa**	**14.8(3)**	**17.5(4)**	**22.2(4)**	**26.9(4)**	**31.1(4)**	**35.3(4)**	**35.3(4)**	**39.2(4)**	**44.1(6)**	**56.6(6)**
***d*(Fe1−O1) x2 (Å)**	2.034(8)	2.033(7)	2.016(12)	2.015(14)	1.997(13)	2.002(19)	2.02(2)	1.98(3)	1.83(11)	2.12(8)
***d*(Fe1−O3) x4 (Å)**	2.124(4)	2.108(4)	2.089(6)	2.083(7)	2.058(7)	2.042(10)	2.050(10)	2.022(13)	2.05(4)	1.98(3)
***d*(Fe2−O2) x4 (Å)**	2.029(6)	2.037(5)	2.022(8)	2.015(9)	1.996(9)	1.986(13)	1.998(14)	2.008(17)	1.99(7)	1.97(6)
***d*(Fe2−O3) x2 (Å)**	1.981(5)	1.963(5)	1.956(7)	1.951(8)	1.939(8)	1.933(11)	1.950(11)	1.920(15)	1.93(4)	1.83(3)
***d*(Fe3−O1) (Å)**	1.920(6)	1.905(5)	1.906(9)	1.878(10)	1.871(9)	1.853(14)	1.842(14)	1.87(2)	1.94(10)	1.76(4)
***d*(Fe3−O2) (Å)**	2.203(5)	2.183(5)	2.165(8)	2.145(8)	2.139(8)	2.129(12)	2.143(12)	2.111(15)	2.15(6)	1.94(4)
***d*(Fe3−O2) x2 (Å)**	2.048(5)	2.026(4)	2.026(7)	2.013(8)	2.006(8)	2.002(12)	1.985(12)	1.980(15)	1.96(6)	1.93(5)
***d*(Fe3−O3) x2 (Å)**	2.025(5)	2.026(5)	2.019(8)	1.998(9)	1.994(9)	1.991(14)	1.975(13)	2.001(18)	1.95(5)	2.00(4)

[1] D. J. Frost, B. T. Poe, R. G. Trønnes, C. Liebske, A. Duba, D. C. Rubie, Phys. Earth Planet. Inter. **143-144**, 507 (2004).

[2] S. V. Ovsyannikov, M. Bykov, E. Bykova, D. P. Kozlenko, A. A. Tsirlin, A. E. Karkin, V. V. Shchennikov, S. E. Kichanov, H. Gou, A. M. Abakumov, R. Egoavil, J. Verbeeck, C. McCammon, V. Dyadkin, D. Chernyshov, S. van Smaalen, and L. S. Dubrovinsky, Nature Chem. **8**, 501 (2016).

[3] S. V. Ovsyannikov, H. Gou, A. E. Karkin, V. V. Shchennikov, R. Wirth *at al.*, Chem. Mater. **26**, 5274 (2014); S. V. Ovsyannikov, E. Bykova, A. Pakhomova, D. P. Kozlenko, M. Bykov *et al.*, Inorg. Chem. **56**, 6251 (2017).

[4] S. V. Ovsyannikov, M. Bykov, E. Bykova, K. Glazyrin, R. S. Manna, A. A. Tsirlin, V. Cerantola, I. Kupenko, A. V. Kurnosov, I. Kantor, A. S. Pakhomova, I. Chuvashova, A. I. Chumakov, R. Rüffer, C. McCammon, and L. S. Dubrovinsky, Nat. Comm. **9**, 4142 (2018).

[5] R. Letoullec, J. P. Pinceaux, and P. Loubeyre, High Press. Res. **1**, 77 (1988).

[6] Y. Fei, A. Ricolleau, M. Frank, K. Mibe, G. Shen, and V. Prakapenka, Proc. Natl. Acad. Sci. U.S.A. **104**, 9182 (2007). .

[7] CrysAlisPro Software system, version 1.171.40.84a, Rigaku Oxford Diffraction, Oxford, UK (2019).

[8] V. Petricek, M. Dusek, and L. Palatinus, Z. Krist. **229**, 345 (2014).

[9] B. Lavina, P. Dera, R. T. Downs, W. Yang, S. Sinogeikin, Y. Meng, and D. Schiferl, Phys. Rev. B **82**, 064110 (2010).

[10] C. Prescher and V. B. Prakapenka, High Press. Res. **35**, 223 (2015).

[11] B. H. Toby and R. B. Von Dreele, J. Appl. Cryst. **46**, 544 (2013).

[12] B. Lavina, P. Dera, E. Kim, Y. Meng, R. T. Downs, P. F. Weck, S. R. Sutton, Y. Zhao, Proc. Natl. Acad. Sci. U.S.A. **108**, 17281 (2011).

[13] G. M. Sheldrick, Acta Crystallogr. Sect. C Struct. Chem. **71**, 3 (2015).

[14] O. V. Dolomanov, L. J. Bourhis, R. J. Gildea, J. A. KHoward, and H. Puschmann, J. Appl. Crystallogr. **42**, 339 (2009).

[15] I. D. Brown, The Chemical Bond in Inorganic Chemistry: The Bond Valence Model, Oxford University Press, 2002; N. E. Brese and M. O'Keeffe, Acta Cryst. B **47**, 192 (1991).

[16] A. D. Chijioke, W. J. Nellis, A. Soldatov, and I. F. Silvera, J. Appl. Phys. **98**, 114905 (2005).

[17] K. Hikosaka, R. Sinmyo, K. Hirose, T. Ishii, and Y. Ohishi, Am. Mineral. **104**, 1356 (2019).

[18] O. L. Anderson,*Equations of State of Solids for Geophysics and Ceramic Science* (Oxford University Press, Oxford, 1995).

[19] J. Gonzalez-Platas, M. Alvaro, F. Nestola, and R. J. Angel, J. Appl. Crystallogr. **49**, 1377 (2016).

[20] We note that a similar behavior was reported for $PrFeO_3$, in which a transition of Fe ions to the LS state was not completed with a sharp isostructural phase transition around 50 GPa and continued as a sluggish spin crossover at higher pressures. See, e.g., G. Kh. Rozenberg, M. P. Pasternak, W. M. Xu, L. S. Dubrovinsky, S. Carlson, R. D. Taylor, Europhys. Lett. **71**, 228 (2005).

[21] T. Chatterji and T. C. Hansen, J. Phys. Condens. Matter **23**, 276007 (2011).

[22] E. Greenberg, I. Leonov, S. Layek, Z. Konopkova, M. P. Pasternak, L. Dubrovinsky, R. Jeanloz, I. A. Abrikosov, and G. K. Rozenberg, Phys. Rev. X. **8**, 031059 (2018).

[23] I. Leonov, G. K. Rozenberg, and I. A. Abrikosov, npj Comput. Mater. **5**, 90 (2019).

[24] E. Greenberg, W. M. Xu, M. Nikolaevsky, E. Bykova, G. Garbarino, K. Glazyrin, D. G. Merkel, L. Dubrovinsky, M. P. Pasternak and G. Kh. Rozenberg, Phys. Rev. B **95**, 195150 (2017); W. M. Xu, G. Yu. Machavariani, G. Kh. Rozenberg, and M. P. Pasternak, Phys. Rev. B **70**, 174106 (2004).

[25] I. Leonov, Phys. Rev. B **92**, 085142 (2015); I. Leonov, L. Pourovskii, A. Georges, and I. A. Abrikosov, Phys. Rev. B **94**, 155135 (2016); I. Leonov and S. Biermann, Phys. Rev. B **103**, 165108 (2021).

[26] I. Leonov, A. O. Shorikov, V. I. Anisimov, and I. A. Abrikosov, Phys. Rev. B **101**, 245144 (2020).

[27] G. Kh. Rozenberg, W. Xu, and M. P. Pasternak, Zeitschrift für Krist.–Cryst. Mater. **229**, 210 (2014).

[28] A. Georges, G. Kotliar, W. Krauth, and M. Rozenberg, Rev. Mod. Phys. **68**, 13 (1996); G. Kotliar, S. Y. Savrasov, K. Haule, V. S. Oudovenko, O. Parcollet, and C. A. Marianetti, Rev. Mod. Phys. **78**, 865 (2006).

[29] L. V. Pourovskii, B. Amadon, S. Biermann, A. Georges, Phys. Rev. B **76**, 235101 (2007); K. Haule, Phys. Rev. B **75**, 155113 (2007); Amadon, B.; Lechermann, F.; Georges, A.; Jollet,

F.; Wehling, T. O.; Lichtenstein, A. I. Phys. Rev. B **77**, 205112 (2008); M. Aichhorn, L. V. Pourovskii, V. Vildosola, M. Ferrero, O. Parcollet, T. Miyake, A. Georges, and S. Biermann, Phys. Rev. B **80**, 085101 (2009); B. Amadon, J. Phys.: Condens. Matter **24**, 075604 (2012).

[30] H. Park, A. J. Millis, and C. A. Marianetti, Phys. Rev. Lett. **109**, 156402 (2012); H. Park, A. J. Millis, and C. A. Marianetti, Phys. Rev. B **89**, 245133 (2014); X. Liao, V. Singh, and H. Park, Phys. Rev. B **103**, 085110 (2021).

[31] V. I. Anisimov, D. E. Kondakov, A. V. Kozhevnikov, I. A. Nekrasov, Z. V. Pchelkina, J. W. Allen, S.-K. Mo, H.-D. Kim, P. Metcalf, S. Suga, A. Sekiyama, G. Keller, I. Leonov, X. Ren, and D. Vollhardt, Phys. Rev. B **71**, 125119 (2005); G. Trimarchi, I. Leonov, N. Binggeli, Dm. Korotin, and V. I. Anisimov, J. Phys.: Condens. Matter **20**, 135227 (2008).

[32] E. Gull, A. J. Millis, A. I. Lichtenstein, A. N. Rubtsov, M. Troyer, and P. Werner, Rev. Mod. Phys. **83**, 349 (2011).

[33] N. F. Mott, Metal-Insulator Transitions, Taylor & Francis, London, 1990.

[34] M. Imada, A. Fujimori, and Y. Tokura, Rev. Mod. Phys. **70**, 1039 (1998).

[35] J. P. Wright, J. P. Attfield, and P.G. Radaelli, Phys. Rev. Lett. **87**, 266401 (2001); J. P. Wright, J. P. Attfield, and P. G. Radaelli, Phys. Rev. B **66**, 214422 (2002).

[36] I. Leonov, A. N. Yaresko, V. N. Antonov, M. A. Korotin, and V. I. Anisimov, Phys. Rev. Lett. **93**, 146404 (2004); I. Leonov, A. N. Yaresko, V. N. Antonov, J. P. Attfield, and V. I. Anisimov, Phys. Rev. B **72**, 014407 (2005); I. Leonov, A. N. Yaresko, V. N. Antonov, and V. I. Anisimov, Phys. Rev. B **74**, 165117 (2006).

[37] J.-F. Lin, J. Wu, J. Zhu, Z. Mao, A. Said, B. Leu, J. Cheng, Y. Uwatoko, C. Jin and J. Zhou, Sci. Rep. **4**, 06282 (2014); F. Baudelet, S. Pascarelli, O. Mathon, J.-P. Itié, A. Polian, and J. Chervin, Phys. Rev. B **82**, 140412 (2010); K. Chen, F. Baudelet, Y. Mijiti, L. Nataf, A. Di Cicco, Z. Hu, S. Agrestini, A. Komarek, A., M. Sougrati, J. Haines, J. Rouquette, Q. Kong, and T.-C. Weng, J. Phys. Chem. C **123**, 21114 (2019).

[38] J. P. Perdew, K. Burke, M. Ernzerhof, Phys. Rev. Lett. **77**, 3865 (1996).
